# Canagliflozin inhibits interleukin-1β-stimulated cytokine and chemokine secretion in vascular endothelial cells by AMP-activated protein kinase-dependent and -independent mechanisms

**DOI:** 10.1038/s41598-018-23420-4

**Published:** 2018-03-27

**Authors:** Sarah J. Mancini, Daria Boyd, Omar J. Katwan, Anastasiya Strembitska, Tarek A. Almabrouk, Simon Kennedy, Timothy M. Palmer, Ian P. Salt

**Affiliations:** 1 Institute of Cardiovascular and Medical Sciences, College of Medical, Veterinary and Life Sciences, University of Glasgow, Glasgow, G12 8QQ UK; 2 Department of Biochemistry, College of Medicine, University of Diyala, Baqubah, Iraq; 3 Medical School, University of Zawia, Zawia, Libya; 4 School of Pharmacy and Medical Sciences, University of Bradford, Bradford, West Yorkshire BD7 1DP UK

## Abstract

Recent clinical trials of the hypoglycaemic sodium-glucose co-transporter-2 (SGLT2) inhibitors, which inhibit renal glucose reabsorption, have reported beneficial cardiovascular outcomes. Whether SGLT2 inhibitors directly affect cardiovascular tissues, however, remains unclear. We have previously reported that the SGLT2 inhibitor canagliflozin activates AMP-activated protein kinase (AMPK) in immortalised cell lines and murine hepatocytes. As AMPK has anti-inflammatory actions in vascular cells, we examined whether SGLT2 inhibitors attenuated inflammatory signalling in cultured human endothelial cells. Incubation with clinically-relevant concentrations of canagliflozin, but not empagliflozin or dapagliflozin activated AMPK and inhibited IL-1β-stimulated adhesion of pro-monocytic U937 cells and secretion of IL-6 and monocyte chemoattractant protein-1 (MCP-1). Inhibition of MCP-1 secretion was attenuated by expression of dominant-negative AMPK and was mimicked by the direct AMPK activator, A769662. Stimulation of cells with either canagliflozin or A769662 had no effect on IL-1β-stimulated cell surface levels of adhesion molecules or nuclear factor-κB signalling. Despite these identical effects of canagliflozin and A769662, IL-1β-stimulated IL-6/MCP-1 mRNA was inhibited by canagliflozin, but not A769662, whereas IL-1β-stimulated c-jun N-terminal kinase phosphorylation was inhibited by A769662, but not canagliflozin. These data indicate that clinically-relevant canagliflozin concentrations directly inhibit endothelial pro-inflammatory chemokine/cytokine secretion by AMPK-dependent and -independent mechanisms without affecting early IL-1β signalling.

## Introduction

The development of vascular endothelial dysfunction, a key early step in atherogenesis, is associated with elevated circulating levels of interleukin (IL)-1β, tumour necrosis factor-α (TNFα) and IL-6^1^. Indeed, recent phase clinical 3 trials indicate that suppression of IL-1β signalling with the monoclonal antibody canakinumab markedly reduced the risk of major adverse cardiovascular events^2^, highlighting the important role of IL-1β in cardiovascular disease. IL-1β simultaneously activates nuclear factor-κB (NFκB) and c-jun N-terminal kinase (JNK) intracellular signalling pathways in cultured vascular endothelial cells, leading to activation of transcription factor complexes stimulating expression of other pro-inflammatory cytokines such as IL-6, adhesion molecules including intercellular cell adhesion molecule-1 (ICAM-1) and the chemokine MCP-1 (monocyte chemoattractant protein-1)^3,4^. The increased expression of adhesion molecules, chemokines and cytokines recruits circulating leukocytes to the vascular wall, which subsequently differentiate into macrophages and accumulate modified low density lipoproteins, leading to foam cell and atherosclerotic plaque formation^1,4^. IL-1β-stimulated NFκB and JNK activation occurs via a complex signalling mechanism, by which IL-1β binding to the IL-1 receptor stimulates formation of a signalosome including TGFβ-activated kinase-1 (TAK1) and inhibitor of NFκB (IκB) kinase (IKK) in a manner dependent on IL-1 receptor associated kinases (IRAKs)^4,5^. TAK1 stimulation leads to phosphorylation and activation of mitogen-activated protein kinase kinases (MKK4 and MKK7) which phosphorylate and activate JNK^5,6^. In parallel, activated IKK phosphorylates IκBα, targeting it for proteasomal degradation and releasing active NFκB dimers^4,5^. Activated JNK phosphorylates nuclear transcription factor complex components, including c-Jun, JunD and ATF-2 whereas NFκB heterodimers translocate into the nucleus and bind the promoters of target genes, leading to increased expression of pro-inflammatory cytokines, adhesion molecules and chemokines. Identification of novel inhibitory mechanisms that may alleviate the pro-inflammatory actions of IL-1β that contribute to atherogenesis is therefore important for potential new therapeutic strategies.

Inhibitors of sodium-glucose co-transporter 2 (SGLT2) are oral hypoglycaemic agents that act to reduce renal glucose reabsorption, thereby increasing glycosuria and reducing hyperglycaemia^7^. Intriguingly, large trials of the SGLT2 inhibitors empagliflozin and canagliflozin in people with type 2 diabetes at high risk of cardiovascular disease have identified that they convey significant improvements in blood pressure, body weight and cardiovascular risk relative to placebo^8,9^. The cardiovascular actions of SGLT2 inhibitors may not be entirely explained by differences in glycaemia, suggesting other mechanisms may be involved^7,10–12^. Recent studies have reported that administration of SGLT2 inhibitors reduce atheroma burden in atherosclerosis-prone mouse models^13,14^. In addition, several recent studies have reported that administration of SGLT2 inhibitors improves pro-inflammatory IL-6, MCP-1 and ICAM-1 gene expression in blood vessels of rodent models of diabetes^13,15–17^. These vascular effects of systemic SGLT2 inhibitor administration may be secondary to changes in glycaemia, blood pressure or actions on extra-cardiovascular tissues, yet may also reflect a direct action on blood vessels. A few studies have investigated direct effects of SGLT2 inhibitors on cardiovascular tissues, with canagliflozin and phlorizin reported to relax murine pulmonary, but not coronary arteries *ex vivo*^18^. Furthermore, empagliflozin has been reported to improve the reduced viability of human umbilical vein endothelial cells (HUVECs) cultured in high glucose concentrations^17^, without altering basal HUVEC proliferation^14^. It therefore remains uncertain whether SGLT2 inhibitors have beneficial direct effects on blood vessels.

We and others have recently demonstrated that clinically-relevant concentrations of canagliflozin, but not dapagliflozin or empagliflozin, activate AMP-activated protein kinase (AMPK) in immortalised cell lines and murine hepatocytes^19,20^. Furthermore, canagliflozin activated hepatic AMPK *in vivo* in mice^19^. Activation of AMPK by canagliflozin was associated with inhibition of complex I of the mitochondrial respiratory chain and increased ADP:ATP ratios^19^, suggesting that canagliflozin activated AMPK through the canonical pathway whereby reduced ATP and increased AMP (or ADP) allosterically activate AMPK, leading to activating phosphorylation of AMPK at Thr172 on the catalytic α subunit by liver kinase B1 (LKB1)^21,22^. Clinically-relevant concentrations of dapagliflozin have recently been reported to stimulate AMPK Thr172 phosphorylation in lipopolysaccharide-stimulated mouse cardiofibroblasts^23^. AMPK is a principal regulator of cellular and whole-body metabolism, yet numerous studies demonstrate that AMPK also regulates multiple pathways in cardiovascular tissues that promote vascular health and inhibit vascular pathology, promoting anti-inflammatory and anti-atherogenic actions in blood vessels^22^. AMPK activation has been demonstrated to suppress palmitate- and TNFα-stimulated NFκB activity in human endothelial cells^24^, likely due to reduced IKKβ activity^25^. Furthermore, AMPK-dependent inhibition of adhesion molecule and MCP-1 expression has also been described in human endothelial cells^25,26^. Furthermore, we have previously demonstrated that direct activation of AMPK with A769662 inhibits IL-1β signalling in murine 3T3-L1 adipocytes and embryonic fibroblasts, reducing IRAK4 autophosphorylation, the phosphorylation of MKK4, JNK, IKK and NFκB translocation to the nucleus^27^.

Since we and others have identified anti-inflammatory actions of AMPK activation in vascular cells and systemic delivery of SGLT2 inhibitors has been reported to impair vascular inflammatory signalling and reduce atheroma in mice, we examined whether SGLT2 inhibitors influence AMPK activity to influence pro-inflammatory signalling pathways in cultured human endothelial cells.

## Results

### Canagliflozin stimulates AMPK in human endothelial cells

**Figure 1:**
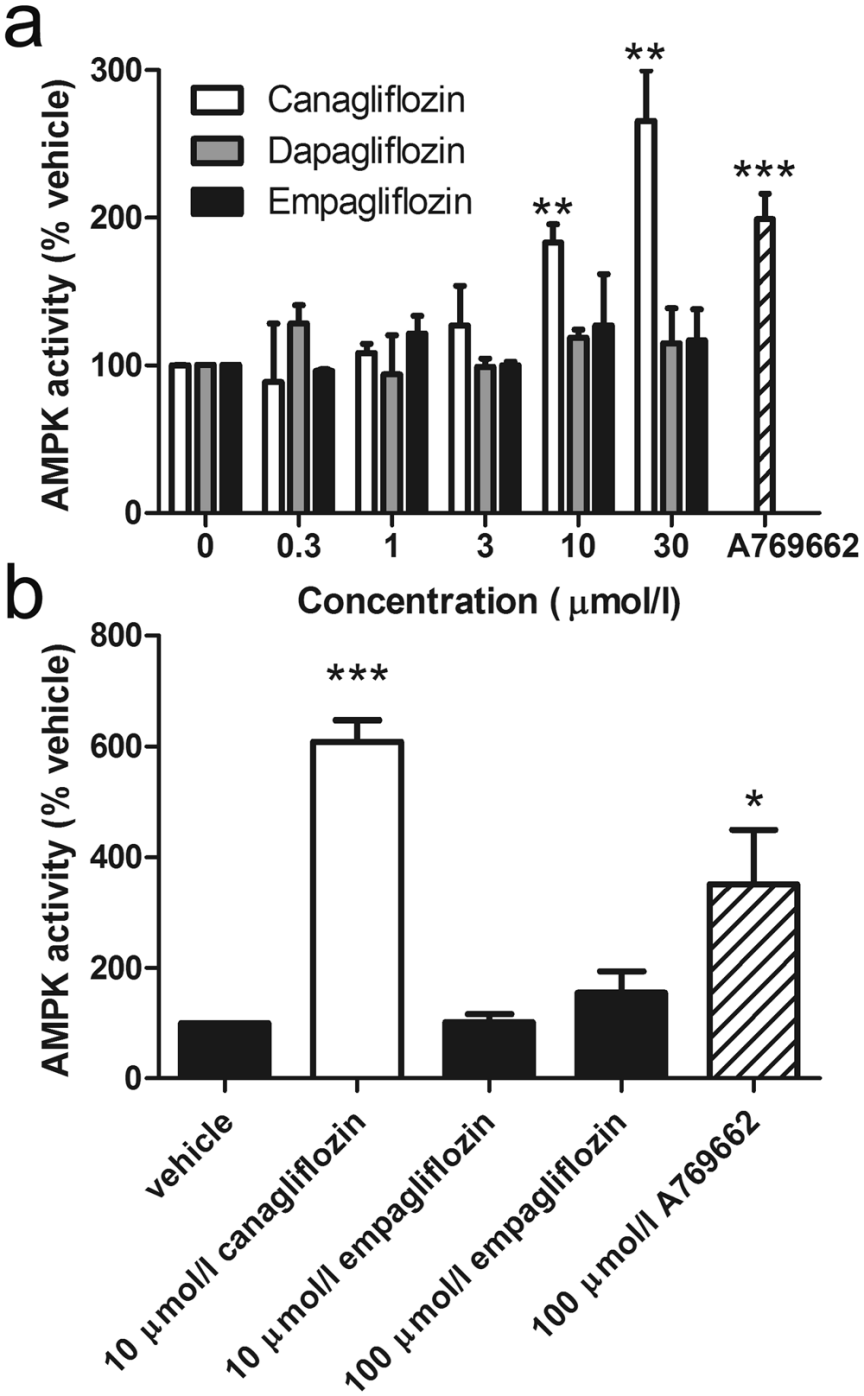
Canagliflozin stimulates AMPK in human endothelial cells. (**a**) HUVECs were stimulated with A769662 (100 μmol/l, 30 min) or the indicated concentrations of canagliflozin, dapagliflozin or empagliflozin for 15 min and lysates prepared. (**b**) HAECs were stimulated with the indicated concentrations of canagliflozin, empagliflozin or A769662 for 30 min and lysates prepared. AMPK was immunoprecipitated from lysates and assayed for AMPK activity. Data shown represents AMPK activity (% vehicle) from three independent experiments. **p < 0.01; ***p < 0.001 vs vehicle (two-tail t-test).

Stimulation of HUVECs with 10 μmol/l canagliflozin significantly stimulated AMPK activity 1.8-fold, similar to AMPK activity in HUVECs stimulated with the direct AMPK activator, A769662 (Fig. 1a). The extent of AMPK activation was further stimulated by increasing the canagliflozin concentration to 30 μmol/l, whereas stimulation of HUVECs with concentrations of dapagliflozin and empagliflozin up to 30 μmol/l had no effect on AMPK activity (Fig. 1a). Activation of AMPK activity was also observed with 10 μmol/l canagliflozin in human aortic endothelial cells (HAECs), whereas concentrations of empagliflozin up to 100 μmol/l had no effect, demonstrating that this effect was not limited to endothelial cells from particular blood vessels (Fig. 1b). Canagliflozin also stimulated AMPK within 30 min in human vascular smooth muscle cells (HAoVSMCs), as assessed by levels of phosphorylation of the AMPK substrate acetyl CoA carboxylase (ACC), demonstrating that canagliflozin-stimulated AMPK activation occurs in multiple vascular cell types (Supplementary Figure S1). Therapeutic doses of canagliflozin reach a peak concentration of approximately 6–8 μmol/l in the plasma of people with diabetes and healthy individuals, whereas dapagliflozin and empagliflozin produce peak concentrations of approximately 1–2 μmol/l^28–30^. As 10 μmol/l canagliflozin and 1 μmol/l empagliflozin or dapagliflozin reflect therapeutically-relevant concentrations, these were used for subsequent experiments.

**Figure 2:**
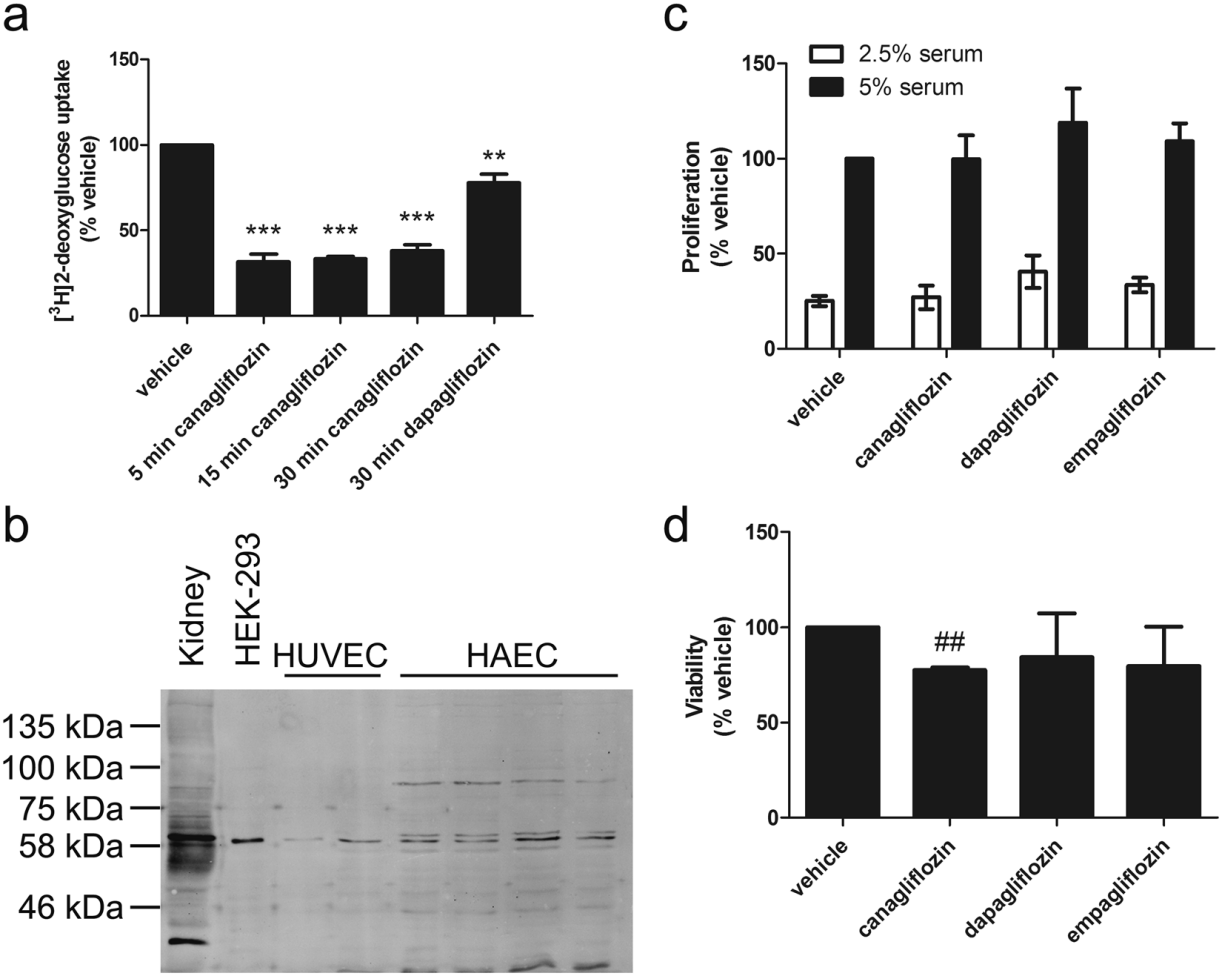
Canagliflozin inhibits 2-deoxyglucose uptake in HUVECs without influencing cell proliferation. (**a**) HUVECs were preincubated in the presence or absence of canagliflozin (10 μmol/l) for the times indicated or dapagliflozin (10 μmol/l, 30 min) and 2-deoxyglucose uptake assessed. Data shown represents the mean 2-deoxyglucose uptake relative to vehicle from three independent experiments. **p < 0.01, ***p < 0.001 relative to vehicle (one-way ANOVA). (**b**) Cell lysates from HUVECs (2 independent lysates), HAECs (4 independent lysates) and HEK-293 cells and mouse kidney membranes were resolved by SDS-PAGE and immunoblotted with anti-SGLT2 antibodies. The immunoblot shown has been cropped, with the full-length immunoblot shown in Supplementary Figure S7. (**c**,**d**) HUVECs were incubated in the presence or absence of canagliflozin, dapagliflozin or empagliflozin and (**c**) proliferation assessed in 2.5% (v/v) or 5% (v/v) MV2 Supplement mix (serum) by BrdU incorporation or (**d**) viability assessed by MTS assay. Data shown represents the mean proliferation or viability relative to vehicle (in 5% MV2 supplement mix) from three independent experiments in each cae. ^##^p < 0.01 relative to vehicle (two-tailed t-test).

We have previously demonstrated that canagliflozin inhibits glucose uptake in mouse embryonic fibroblasts and HEK-293 cells^19^. Canagliflozin rapidly inhibited 2-deoxyglucose uptake by approximately 70% in HUVECs, whereas dapagliflozin caused a more modest inhibition of 2-deoxyglucose uptake (~25%) (Fig. 2a). Immunoreactive bands of approximately 60 kDa could be detected with anti-SGLT2 antibodies in HUVEC and HAEC cell lysates that co-migrated with bands in the human embryonic kidney cell line HEK-293 and a mouse kidney membrane fraction (Fig. 2b). Despite this and the inhibition of 2-deoxyglucose uptake by canagliflozin and dapagliflozin, SGLT2 mRNA was undetectable in HAECs or HUVECs, as well in the human embryonic kidney cell line HEK-293, whereas SGLT2 mRNA was detectable in cDNA prepared from human kidney cortex (Table 1). Although canagliflozin and dapagliflozin are selective inhibitors of SGLT2, the concentrations used could also inhibit SGLT1^31^.

**Table 1:** Expression of SGLT2 and SGLT1 mRNA in human endothelial cells. Expression of SGLT2, SGLT1 relative to TBP was assessed by qPCR in cDNA prepared from three independent cultures of HUVECs, HAECs and HEK-293 cells, using cDNA from human kidney cortex as a positive control. Data shown are the mean ± SEM Ct values and 2^−ΔΔCt^ in each case, from three independent samples. ND = Not detected.

Sample	Ct (TBP)	Ct (SGLT1)	SGLT1 2^−ΔΔCt^	Ct (SGLT2)	SGLT2 2^−ΔΔCt^
Human kidney cortex	25.00	22.68	1	17.73	1
HAEC	23.67 ± 0.39	34.65 ± 0.23	1.04 × 10^−4^ ± 0.22 × 10^−4^	ND	ND
HUVEC	23.53 ± 0.12	34.24 ± 0.29	1.26 × 10^−4^ ± 0.28 × 10^−4^	ND	ND
HEK-293	22.10 ± 0.16	30.89 ± 0.45	4.61 × 10^−4^ ± 0.94 × 10^−4^	ND	ND

Specific anti-SGLT1 immunoreactivity could not be demonstrated in lysates from HUVEC, HAEC and HEK-293 cells or a mouse kidney membrane fraction, as several immunoreactive bands were detected with anti-SGLT1 antibodies (Supplementary Figure S2). Similarly, SGLT1 mRNA was expressed at very low levels in HAECs, HUVECs or HEK-293 cells, yet showed significant expression in kidney cortex (Table 1). HUVEC proliferation was stimulated by increasing the concentration of MV2 supplement mix, which contains serum and growth factors, from 2.5% − 5% (v/v) (Fig. 2c). Despite the inhibition of 2-deoxyglucose uptake, however, incubation with canagliflozin, dapagliflozin or empagliflozin for 36 h had no effect on HUVEC proliferation in either concentration of supplement mix (Fig. 2c), although canagliflozin did modestly reduce HUVEC viability as assessed with a tetrazolium-based assay (Fig. 2d).

### Canagliflozin inhibits IL-1β-stimulated secretion of MCP-1 and IL-6 in an AMPK-dependent manner, associated with reduced mRNA expression

**Figure 3:**
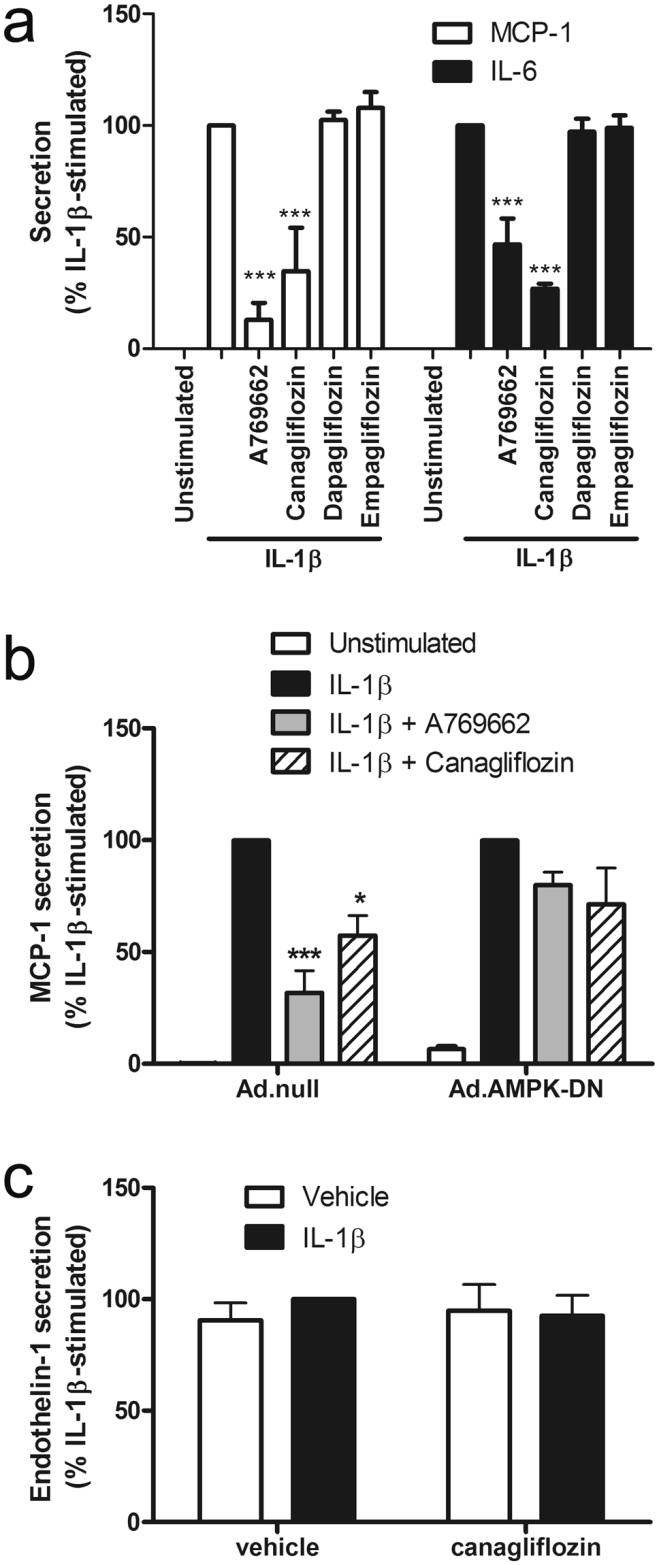
Canagliflozin inhibits IL-1β-stimulated MCP-1 and IL-6 secretion in human endothelial cells. (**a**,**c**) HUVECs were stimulated with IL-1β (10 ng/ml) for (**a**) 6 h or (**c**) 24 h following preincubation in the presence or absence of canagliflozin (10 μmol/l, 15 min), dapagliflozin (1 μmol/l, 15 min), empagliflozin (1 μmol/l, 15 min) or A769662 (100 μmol/l, 30 min) and conditioned medium collected. (**b**) HAECs were infected with 100 pfu/cell Ad.null or Ad.AMPK-DN for 24 h and preincubated in the presence or absence of canagliflozin (10 μmol/l, 15 min) or A769662 (100 μmol/l, 30 min) prior to stimulation with IL-1β (10 ng/ml) for 6 h and conditioned medium collected. (**a**,**b**) MCP-1, (**a**) IL-6 or (**c**) endothelin-1 levels were assayed in conditioned medium by ELISA. Data shown represents the % IL-1β-stimulated MCP-1, IL-6 or endothelin-1 secretion from (**a**,**c**) three (**b**) four (Ad.null) or five (Ad.AMPK-DN) independent experiments. *p < 0.05, **p < 0.01, ***p < 0.001 relative to IL-1β alone (ANOVA).

**Figure 4:**
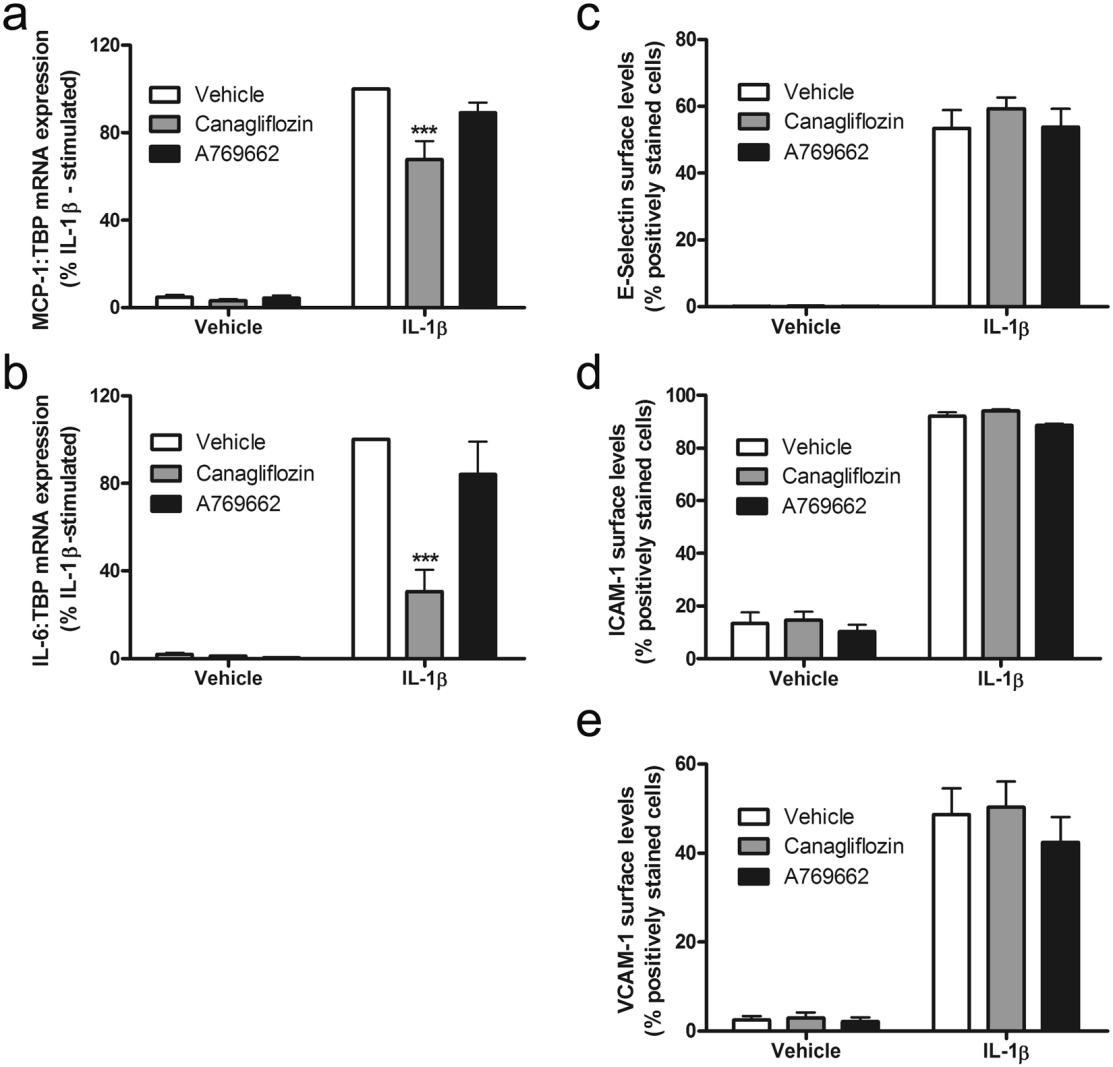
Canagliflozin suppresses IL-1β-stimulated MCP-1 and IL-6 mRNA expression, without altering cell surface adhesion molecule levels in HAECs. HAECs were stimulated with (**a**,**b**) IL-1β (10ng/ml) for 6 h or (**c**,**d**,**e**) IL-1β (5 ng/ml) for 4 h following preincubation in the presence or absence of A769662 (30 min, 100 μmol/l) or canagliflozin (15 min, 10 μmol/l). (**a**) MCP-1 or (**b**) IL-6 mRNA levels were analysed by qPCR and cell surface levels of (**c**) E-selectin, (**d**) ICAM-1 or (**e**) VCAM-1 were assessed by flow cytometry. Data shown represents (a, b) % IL-1β-stimulated mRNA expression normalised to TBP from four independent experiments or (**c**,**d**,**e**) % positively stained HAECs from three independent experiments. ***p < 0.001 relative to IL-1β alone (ANOVA).

Canagliflozin inhibited IL-1β-stimulated MCP-1 and IL-6 secretion by HUVECs to a similar extent to the inhibition by A769662 (Fig. 3a), whereas neither dapagliflozin nor empagliflozin had any effect, indicating that canagliflozin-stimulated AMPK activation may underlie this anti-inflammatory action. The anti-inflammatory action of canagliflozin was not limited to HUVECs, as it also inhibited IL-1β-stimulated MCP-1 secretion in human aortic endothelial cells (HAECs) (Fig. 3b). Furthermore, the inhibitory effect of canagliflozin was likely to be AMPK-dependent, as infection of HAECs with adenoviruses expressing a dominant negative mutant AMPKα1 (Ad.AMPK-DN)^32^ attenuated the effect of canagliflozin and A769662 (Fig. 3b). Stimulation of AMPK was still observed after 6 h incubation with canagliflozin or A769662, as assessed by phosphorylation of the AMPK substrate ACC and infection with Ad.AMPK-DN markedly suppressed canagliflozin- and A769662-stimulated ACC phosphorylation (Supplementary Figure S3). Canagliflozin did not have a general effect on secretion, as canagliflozin had no effect on endothelin-1 secretion by HUVECs, which was not stimulated by IL-1β (Fig. 3c). Canagliflozin inhibited IL-1β-stimulated expression of MCP-1 and IL-6 mRNA in HAECs, suggesting that this may underlie, at least in part, the inhibition of IL-1β-stimulated MCP-1/IL-6 secretion (Fig. 4a,b). In contrast, incubation with A769662 did not significantly inhibit expression of either MCP-1 or IL-6 mRNA, indicating that the effect of canagliflozin on MCP-1 or IL-6 mRNA expression is AMPK-independent.

### Canagliflozin and AMPK activators inhibit IL-1β-stimulated U937 cell adhesion to endothelial cells without altering cell surface adhesion molecule levels

**Figure 5:**
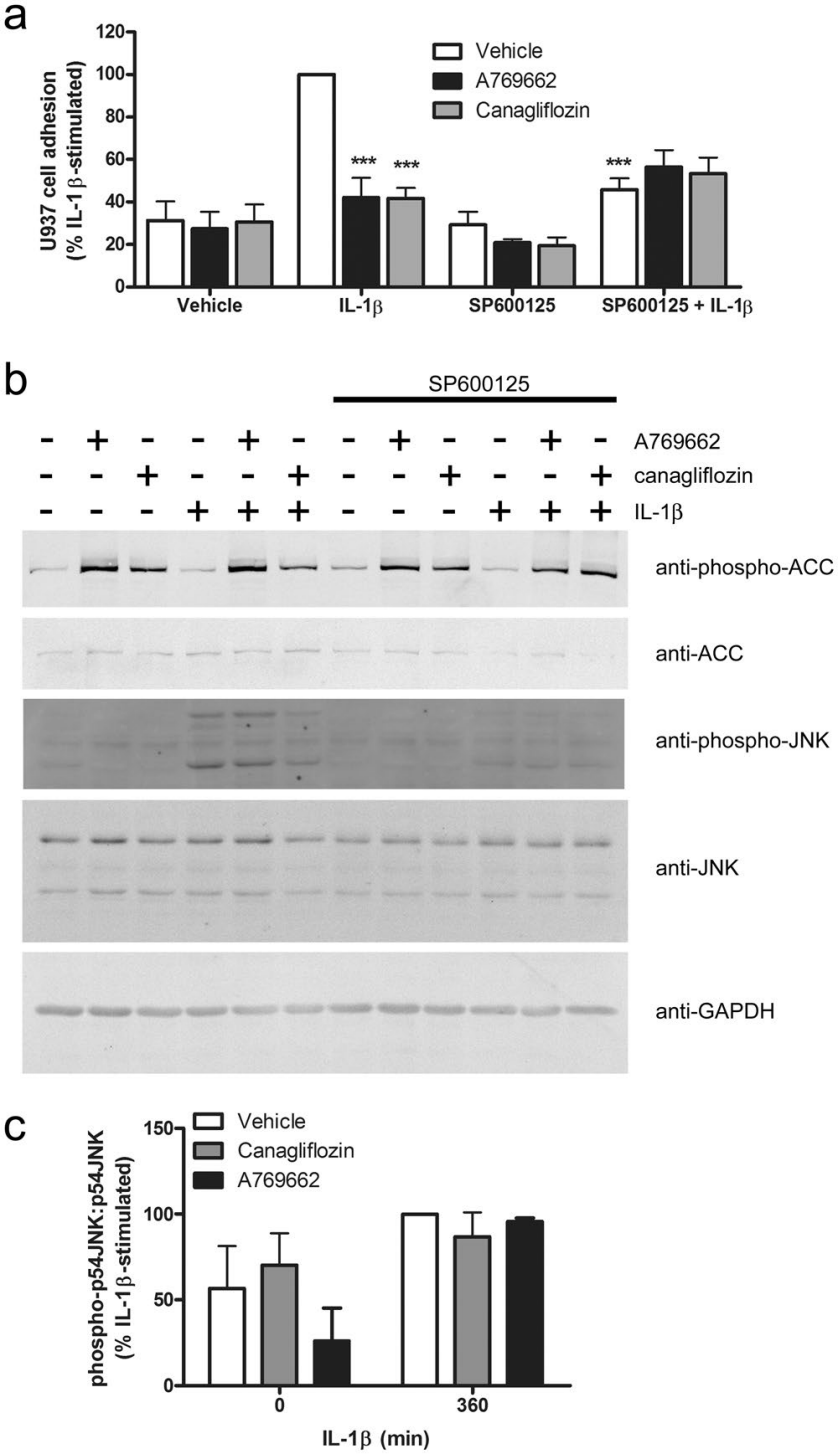
Canagliflozin inhibits IL-1β-stimulated adhesion of U937 cells to HUVECs. HUVECs were stimulated in the presence or absence of SP600125 (60 min, 30 μmol/l), A769662 (30 min, 100 μmol/l) or canagliflozin (15 min, 10 μmol/l) prior to stimulation with IL-1β (10 ng/ml) for 6 h. and (**a**) U937 cell adhesion assessed or (**b**) lysates prepared. (**a**) Data shown represents the % IL-1β-stimulated U937 cell adhesion from three independent experiments, with 8 fields of cells examined in each technical replicate. Representative micrographs are shown in Supplementary Figure S8. (**b**,**c**) HUVEC lysates were immunoblotted with the antibodies indicated. (**b**) Representative images are shown and have been cropped, with the full-length immunoblots shown in Supplementary Figure S9. (**c**) Densitometric analysis of JNK (p54) phosphorylation normalised to respective total JNK from four (canagliflozin) or two (A769662) independent experiments. ***p < 0.001 relative to IL-1β alone (ANOVA).

We have previously demonstrated that brief preincubation with AMPK activators inhibits TNFα-stimulated U937 promonocytic cell adhesion to and MCP-1 secretion by HAECs prior to any changes in cell surface adhesion molecule expression^26,33^. In agreement with this, neither canagliflozin nor A769662 inhibited IL-1β-stimulated cell surface expression of E-selectin, vascular cell adhesion molecule-1 (VCAM-1) or intercellular adhesion molecule-1 (ICAM-1) (Fig. 4c–e), yet both canagliflozin and A769662 markedly inhibited IL-1β-stimulated U937 cell adhesion (Fig. 5a), without altering basal U937 cell adhesion.

### A769662, but not canagliflozin inhibits rapid IL-1β-stimulated JNK phosphorylation

**Figure 6:**
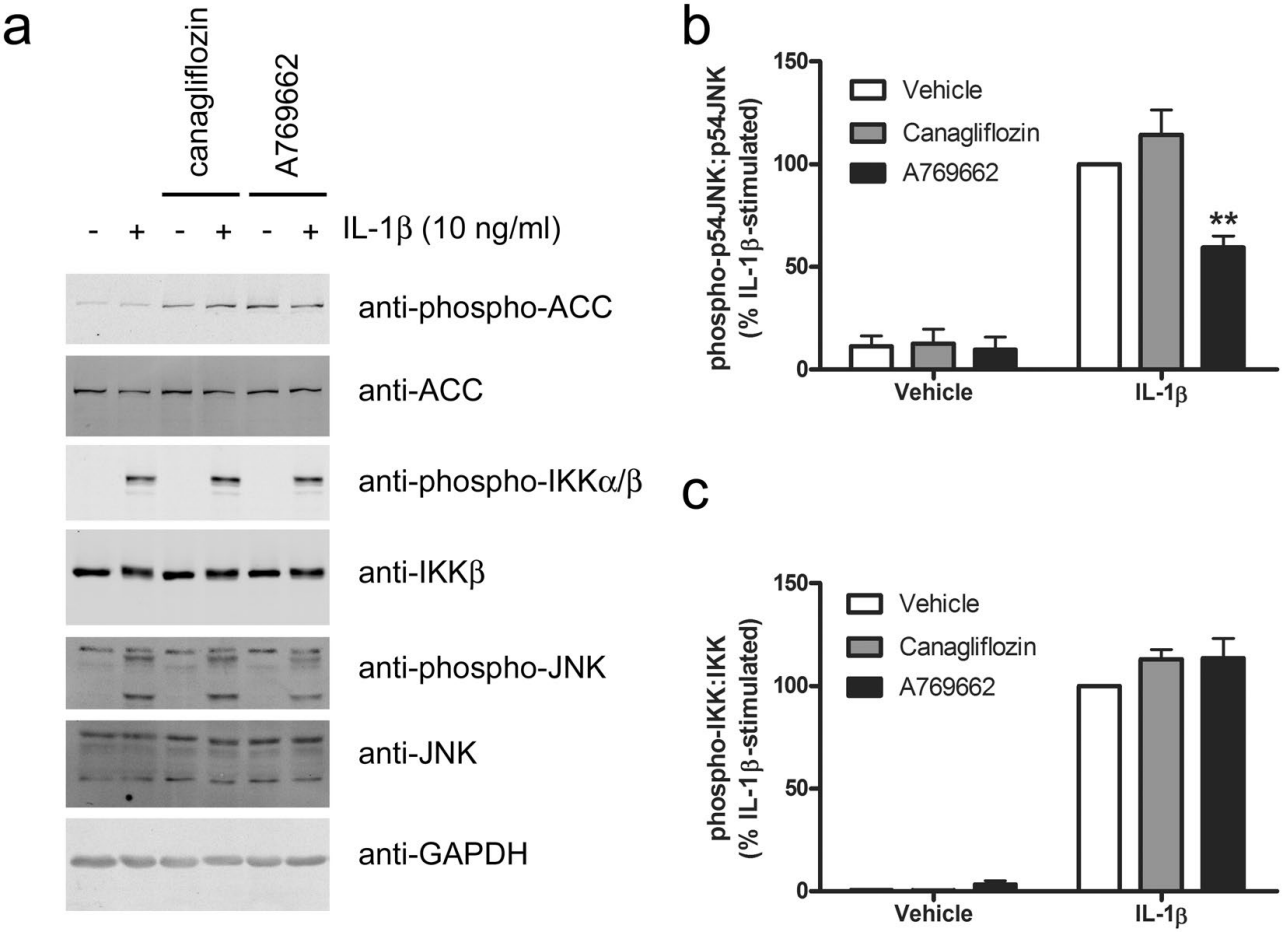
A769662 but not canagliflozin inhibits early IL-1β-stimulated JNK phosphorylation. HUVECs were incubated in the presence or absence of canagliflozin (10 μmol/l, 15 min) or A769662 (100 μmol/l, 30 min) prior to stimulation with IL-1β (10 ng/ml, 15 min) and lysates prepared. Lysate proteins were resolved by SDS-PAGE and subjected to immunoblotting with the antibodies indicated. (**a**) Representative immunoblots, repeated on two further occasions with similar results are shown and have been cropped, with the full-length immunoblots shown in Supplementary Figure S10. (**b**,**c**) Densitometric analysis of (**b**) JNK (p54) and (**c**) IKK phosphorylation normalised to respective total levels from three independent experiments. **p < 0.01 vs absence of A769662 (ANOVA).

**Figure 7:**
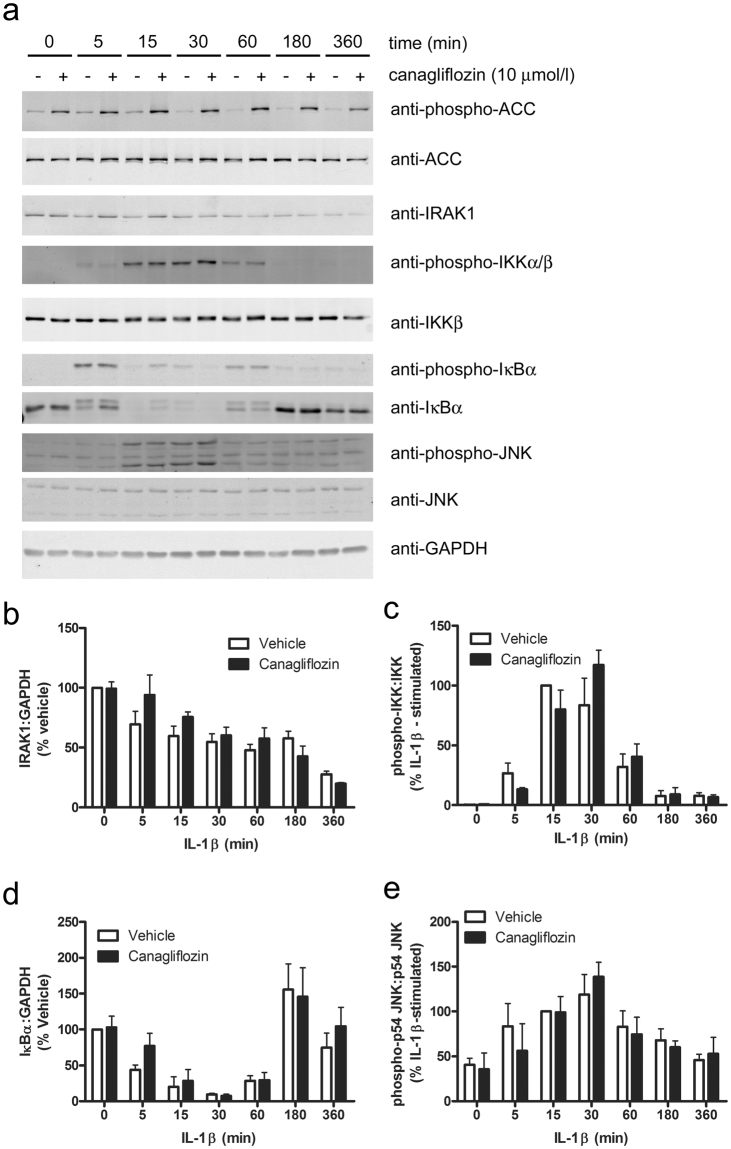
Effect of 10 μmol/l canagliflozin on initial IL-1β pro-inflammatory signalling pathways. HUVECs were incubated for 15 min in the presence or absence of canagliflozin (10 μmol/l) prior to stimulation with IL-1β (10 ng/ml) for the times indicated and lysates prepared. Lysate proteins were resolved by SDS-PAGE and subjected to immunoblotting with the antibodies indicated. (**a**) Representative images are shown and have been cropped, with the full-length immunoblots shown in Supplementary Figure S11. (**b**–**e**) Densitometric analysis of (**b**) IRAK1, (**d**) IκB relative to GAPDH (% vehicle) or (**c**) IKK, (**e**) JNK (p54) phosphorylation (% 15 min IL-1β-stimulated) normalised to respective total levels from three independent experiments.

We have recently reported that A769662 inhibited IL-1β-stimulated JNK phosphorylation, IKK phosphorylation and p65 NFκB translocation to the nucleus in 3T3-L1 adipocytes and mouse embryonic fibroblasts in an AMPK-dependent manner^27^. We therefore investigated the role of JNK and NFκB in the inhibition of the actions of IL-1β. Incubation of HUVECs with the JNK-selective inhibitor SP600125 phenocopied the effect of canagliflozin on U937 cell adhesion (Fig. 5a), without altering canagliflozin- or A769662-stimulated AMPK activity, as assessed by ACC phosphorylation, which remained elevated after 6 h stimulation (Fig. 5b). Despite this, neither canagliflozin nor A769662 had any significant effect on levels of phosphorylated JNK after 6 h stimulation with IL-1β (Fig. 5b,c). In contrast to the lack of effect on levels of phosphorylated JNK after 6 h stimulation with IL-1β, A769662 did inhibit the initial IL-1β-stimulated JNK phosphorylation within 15 min, yet canagliflozin had no effect (Fig. 6). Similarly, canagliflozin had no significant effect on IL-1β-stimulated JNK phosphorylation in HAoVSMCs and stimulation of HAoVSMCs with the alternative pro-inflammatory stimulus angiotensin II had no effect on JNK phosphorylation (Supplementary Figure S1). Stimulation of HUVECs with an alternative direct AMPK activator, compound 991, which is reported to bind to the same site on AMPK as A769662^34^, markedly increased ACC phosphorylation in HUVECs and inhibited IL-1β-stimulated JNK phosphorylation (Supplementary Figure S4). Conversely, infection of HAECs with Ad.AMPK-DN markedly increased IL-1β-stimulated phosphorylation of JNK (Supplementary Figure S4). Despite our previous observation that A769662 inhibited NFκB signalling in 3T3-L1 adipocytes and mouse embryonic fibroblasts^27^, neither A769662 nor canagliflozin had any effect on IL-1β-stimulated IKK phosphorylation (Fig. 6) or p65 NFκB translocation to the nucleus after 15 min in HUVECs (Supplementary Figure S5), and canagliflozin had no effect on IL-1β-stimulated IKK or IκBα phosphorylation or degradation of IRAK1 or IκBα at any time point examined (Fig. 7). Increasing the canagliflozin concentration to 30 μmol/l did, however, cause a modest yet significant reduction in early (5 min) IL-1β-stimulated IKK and JNK phosphorylation, as well as IκB degradation (Supplementary Figure S6).

## Discussion

The principal finding of this study is that clinically-relevant concentrations of canagliflozin, but not dapagliflozin or empagliflozin inhibit IL-1β-stimulated secretion of the key pro-inflammatory cytokine IL-6 and chemokine MCP-1 in cultured human endothelial cells. Furthermore, we show that this effect of canagliflozin is, at least in part, AMPK-dependent and associated with reduced adhesion of pro-monocytic cells. However, canagliflozin has no significant effect on IL-1β-stimulated JNK or NFκB signalling, suggesting the effect on chemokine/cytokine secretion and monocyte adhesion is independent of early pro-inflammatory signalling. Comparison of the effects of canagliflozin with the direct AMPK activator, A769662, also suggested that canagliflozin inhibits IL-1β-stimulated MCP-1 and IL-6 gene expression in an AMPK-independent manner. These data suggest that canagliflozin could suppress vascular inflammatory signalling, which is associated with the development of cardiovascular disease.

The finding that only canagliflozin of the three SGLT2 inhibitors examined activated AMPK in cultured human endothelial cells is in agreement with our and others’ previously published reports in immortalised cell lines and murine hepatocytes^19,20^. Furthermore, we show that the same clinically-relevant concentration of canagliflozin activates AMPK in HAoVSMCs. We have previously reported that canagliflozin inhibits complex I in the mitochondrial respiratory chain, which is likely to underlie the AMPK activation due to increased AMP:ATP^19^. In the current study we did not determine mitochondrial function, yet canagliflozin did modestly reduce HUVEC viability without altering proliferation as assessed with an MTS-based measurement that reflects levels of NAD(P)H produced by dehydrogenase enzymes in metabolically active cells^35^, such that the canagliflozin-mediated reduction may indicate reduced mitochondrial dehydrogenase activity. Canagliflozin, and to a lesser extent dapagliflozin, inhibited HUVEC 2-deoxyglucose uptake, as we have previously observed in HEK-293 cells and mouse embryonic fibroblasts^19^. It is feasible that reduced glucose uptake could impair ATP synthesis and thereby contribute to the activation of AMPK by canagliflozin. Importantly, the medium (MV2) used in the majority of the experiments described also contains amino acids, which would be predicted to provide an alternative source of ATP in the event of inhibited glucose transport and phosphorylation. It therefore remains unclear whether reduced glycolytic flux from glucose synergises with the inhibition of mitochondrial complex I that we have previously reported^19^ to impair ATP production and stimulate AMPK in response to canagliflozin in endothelial cells.

Although canagliflozin and dapagliflozin are selective inhibitors of SGLT2, the concentrations used could also inhibit SGLT1^31^. In agreement with previous studies in human coronary or pulmonary artery endothelial cells^18^, SGLT2 mRNAs were expressed at undetectable levels in HUVECs and HAECs. Despite the lack of SGLT2 mRNA, immunoreactive bands of approximately 60 kDa were detected with anti-SGLT2 antibodies in endothelial cell lysates that co-migrated with a band in mouse kidney membranes, so it remains uncertain whether SGLT2 protein is present in human endothelial cells and underlies the inhibition of 2-deoxyglucose uptake by canagliflozin. SGLT1 mRNAs were expressed at very low levels in HUVECs and HAECs, similar to a previous report^36^, yet we were unable to detect SGLT1 protein by immunoblotting, unlike a previous study in HUVECs^37^. The inhibition of 2-deoxyglucose uptake in HUVECs by 10 μmol/l canagliflozin may therefore be due to inhibition of a facilitative glucose transporter, as has been suggested before in a rat skeletal muscle cell line^38^.

Two recent studies have indicated that dapagliflozin may also stimulate AMPK activity. Administration of dapagliflozin for eight weeks increased cardiac AMPK Thr172 phosphorylation in lipodystrophic seipin knockout mice^39^ and stimulation of murine LPS-stimulated cardiofibroblasts with 0.4 μmol/l dapagliflozin for 16 h stimulated AMPK phosphorylation, associated with reduced intracellular NLRP3 inflammasome and TNFα levels^23^. As these latter effects were mimicked by A769662 and inhibited by compound C, the authors argue the effect of dapagliflozin is AMPK-mediated, although it should be noted that compound C inhibits several other protein kinases with greater efficacy than AMPK^40^. In the current study, 0.3 μmol/l dapagliflozin had no effect on AMPK activity in HUVECs, suggesting the discrepancy between our study and that of Ye and colleagues may either reflect a tissue-specific effect, the LPS-stimulated background utilised by Ye and colleagues, in which AMPK activity is inhibited, or may be due to the markedly different incubation times with SGLT2 inhibitor used.

Several lines of evidence suggest that the marked inhibition of MCP-1 secretion by canagliflozin is AMPK-dependent. Firstly, the inhibition was mimicked by the direct AMPK activator, A769662 and secondly, of the three SGLT2 inhibitors examined, only canagliflozin activated AMPK and only canagliflozin reduced IL-1β-stimulated MCP-1 secretion in both HUVECs and HAECs. Finally, as the canagliflozin-mediated inhibition was attenuated by infection with Ad.AMPK-DN, the inhibitory action of canagliflozin on chemokine/cytokine secretion is at least partially AMPK dependent. In previous studies, we have reported that short-term incubation with AICAR, which is converted to the AMP mimetic ZMP within cells^41^, inhibited TNFα-stimulated MCP-1 secretion by and U937 cell adhesion to HAECs in an AMPK-dependent manner, without altering cell surface adhesion molecule expression^26^. In the current study, stimulation with canagliflozin or A769662 inhibited IL-1β-stimulated U937 cell adhesion without altering cell surface adhesion molecule levels in a similar manner. This further supports an AMPK-dependent mechanism for the inhibition of MCP-1 secretion by canagliflozin that is likely to underlie the inhibition of monocyte adhesion without altering levels of cell surface adhesion molecules.

Despite the similar effects of A769662 and canagliflozin on endothelial cell AMPK activity, IL-1β-stimulated MCP-1/IL-6 secretion, U937 cell adhesion and cell surface adhesion molecule expression, there were some differential actions of canagliflozin and A769662 upstream of the effect on MCP-1 and IL-6 secretion. Canagliflozin inhibited IL-1β-stimulated expression of both MCP-1 and IL-6 mRNA, whereas A769662 had no effect. Given the similar stimulation of AMPK by both compounds, it seems likely, therefore, that this effect of canagliflozin is AMPK-independent. In contrast, A769662 and compound 991 inhibited IL-1β-stimulated JNK phosphorylation, whereas canagliflozin only altered JNK phosphorylation at high concentrations, suggesting that AMPK activation is not sufficient to inhibit JNK phosphorylation. The differential effects of canagliflozin and A769662 may also reflect their different modes of activating AMPK. Whereas canagliflozin increases ADP:ATP, reducing cellular energy charge and allosterically activating AMPK by the canonical mechanism, A769662 and compound 991 directly activate AMPK complexes containing the β1 non-catalytic subunit isoform without altering cellular adenine nucleotide ratios^4,19–22^. As a consequence, it would be predicted that canagliflozin activates all AMPK complexes as well as inhibiting ATP synthesis and thereby other pathways that are sensitive to ATP or AMP concentrations, whereas A769662 (and compound 991) only activate AMPK complexes containing β1 without altering cellular energy charge directly. Indeed, AMPKβ1 and AMPKβ2 have been reported to have markedly different subcellular localisation in HUVECs^42^, suggesting the potential for differential roles. Stimulation of different pools of AMPK and parallel effects on AMPK-independent pathways may, therefore underlie the different effects of canagliflozin compared to A769662 and compound 991 upstream of IL-1β-stimulated monocyte adhesion and secretion of MCP-1 and IL-6.

Given the widely-reported anti-inflammatory actions of AMPK activation in response to several proinflammatory cytokines in endothelial cells^22^, it is tempting to speculate that there is a common anti-inflammatory mechanism of action for AMPK activators, yet cytokines such as IL-1β, TNFα and IL-6 employ different signalling mechanisms, with various degrees of shared intermediates. Multiple mechanisms have been described by which AMPK may inhibit NFκB activation^25,43^, and we have previously reported in murine 3T3-L1 adipocytes and embryonic fibroblasts that A769662 inhibits IL-1β-stimulated JNK and NFκB activation, yet had little effect on TNFα-stimulated JNK phosphorylation^27^. Furthermore, the inhibition by AMPK was associated with inhibition of IRAK4 autophosphorylation, arguing for an inhibitory effect of AMPK on the first steps of IL-1β signalling^27^. In the current study, neither A769662 nor canagliflozin had any effect on IL-1β-stimulated IKKβ phosphorylation or translocation of p65 NFκB to the nucleus, which was surprising given the marked effect of both compounds on MCP-1 secretion and key role NFκB activation has in the stimulation of MCP-1 gene expression. This further indicates that the mechanism by which AMPK activation inhibits IL-6 and MCP-1 secretion in endothelial cells is independent of effects on NFκB signalling and gene expression.

As AMPK activation inhibits the mechanistic target of rapamycin complex 1 (mTORC1), which is a key regulator of translation, this could represent a common target that explains the inhibition of MCP-1 and IL-6 secretion by both canagliflozin and A769662 in the absence of consistent effects on mRNA expression. Indeed, inhibition of mTORC1 has been reported to inhibit IL-6 secretion^44,45^, yet without affecting basal or TNFα-stimulated MCP-1 secretion^44^, suggesting that AMPK-mediated mTORC1 suppression is unlikely to mediate the effects observed in this study. AMPK activation has also been reported to influence endothelial endocytosis^46^, yet, to our knowledge, there are no reports of AMPK activation having any direct effect on the secretory pathway in endothelial cells. Given that we have also shown that AMPK activation inhibits IL-6-stimulated activation of STAT (signal transducer and activator of transcription) transcription factors^27,47^, likely to be mediated by AMPK-mediated phosphorylation and inhibition of Janus kinase 1^47^, it is entirely likely that AMPK activation inhibits multiple pro-inflammatory pathways by multiple mechanisms that are cell-specific, such that care should be taken when examining the effects of AMPK activation on pro-inflammatory signalling in different tissues and species.

Previous studies have reported that systemic administration of the SGLT2 inhibitors empagliflozin, ipragliflozin or luseogliflozin decreases pro-inflammatory IL-6, MCP-1 and ICAM-1 gene expression in blood vessels of rodent models of diabetes^13,15–17^ and canagliflozin administration inhibits inflammation in metabolic tissues of high fat diet-fed mice^48^. The reduced vascular inflammatory signalling observed with other SGLT2 inhibitors could be direct, but may also be secondary to changes in blood glucose concentrations, blood pressure and the effects on metabolic tissues such as the liver or adipose tissue over time. To our knowledge, no previous study has examined the direct actions of SGLT2 inhibitors on indices of vascular inflammation, such that our data suggests canagliflozin alone among the three SGLT2 inhibitors we examined has the potential to have a direct effect on vascular cells. It does remain possible, however, that prolonged incubation, rather than the acute stimulations we have used, of human endothelial cells with dapagliflozin or empagliflozin may inhibit pro-inflammatory stimuli or indeed activate AMPK.

In summary, canagliflozin inhibits IL-1β-stimulated secretion of the key pro-inflammatory, pro-atherogenic mediators MCP-1 and IL-6 in an AMPK-dependent manner, yet the precise mechanism(s) underlying this remains elusive. These data further suggest that clinically-relevant concentrations of canagliflozin have the potential to directly influence blood vessels in people with type 2 diabetes as well as lower blood glucose concentrations. It is tempting to speculate that the anti-inflammatory actions of canagliflozin we have identified could have an anti-atherogenic action in blood vessels, yet more research is required to examine the extent to which these might contribute to their beneficial cardiovascular actions in people with type 2 diabetes.

## Materials and Methods

### Materials

Adenoviruses expressing c-myc-tagged dominant negative mutant AMPKα1 (Ad.AMPK-DN) and control adenoviruses (Ad.null) have been described previously^32^, and were a generous gift from Dr F. Foufelle, Centre Biomédical des Cordeliers, Paris. Kidney cortex cDNA was prepared by Dr. Dominik Skiba, University of Glasgow, UK from the healthy margin of renal tumours obtained through the NHSGCC Biorepository and Pathology Service Tissue Resource (approved by West of Scotland Research Ethics Services No 10/S0704/60). Canagliflozin, dapagliflozin and empagliflozin were obtained from Selleckchem (Newmarket, UK). A769662 was obtained from Abcam (Cambridge, UK). [γ-^32^P]-ATP was obtained from Perkin Elmer (Buckinghamshire, UK). SAMS peptide was obtained from GL Biochem Ltd (Shanghai, China). SP600125 was from Bio-Techne (Abingdon, UK). Recombinant human IL-1β and Quantikine ELISA kits for human endothelin-1, IL-6 and MCP-1 were obtained from R&D Systems (Abingdon, UK). Phycoerythrin-conjugated anti-ICAM1 (#555512), anti-VCAM-1 (#561679) and anti-E-selectin (#550040) antibodies were from BD Transduction Labs (Oxford, UK). Rabbit anti-phospho-acetyl CoA carboxylase (ACC) Ser79 (#3661), anti-ACC (#3676), anti-phospho-JNK Thr183/Tyr185 (#4668), anti-JNK (#9252), anti-phospho-IκBα Ser32 (#2859), anti-phospho-IKKα/β Ser176/Ser177 (#2078), anti-IKKβ (#8943), anti-IRAK1 (#4504), anti-α-tubulin (#2125) and mouse anti-IκBα (#4814) antibodies were from New England Biolabs (Hitchin, Hertfordshire, UK). Mouse anti-GAPDH was obtained from Ambion (AM4300) (Cambridge, UK). Goat anti-SGLT2 (sc-47402) antibodies were from Santa Cruz Biotechnology (Santa Cruz, CA, USA). Sheep anti-AMPKα1 and anti-AMPKα2 antibodies for immunoprecipitation kinase assays have been described before^49^ and were a kind gift from Prof. D. G. Hardie, University of Dundee (Dundee, UK). Donkey Infrared dye-labelled secondary antibodies were from LI-Cor Biosciences (Cambridge, UK). CellTiter 96® AQ_ueous_ One solution was purchased from Promega (Southampton, UK). BrdU cell proliferation assay kits were obtained from Millipore (Watford, UK). All other reagents were from sources described previously^26,27,50^.

### Cell culture

Human umbilical vein endothelial cells (HUVECs) and aortic endothelial cells (HAECs) were cultured in MV2 medium (Promocell, Heidelberg, Germany) and used for experiments between passages 3 and 6 as described previously^50^. HEK-293 cells were cultured in DMEM, supplemented with 10% (v/v) foetal calf serum, 1 mmol/l pyruvate and 2 mmol/l L-glutamine as described previously^19^. U-937 pro-monocytic cells were grown in RPMI 1640 medium, supplemented with 10% (v/v) foetal calf serum and 2 mmol/l L-glutamine as described previously^26,33^.

### Preparation of adenoviruses and infection of HAECs

Ad.AMPK-DN and Ad.null were propagated, purified and titred as described previously^26^. HAECs were infected with adenoviruses for 24 h in MV2 medium prior to experimentation.

### Preparation of cell lysates, SDS PAGE and immunoblotting

HUVECs or HAECs were serum-starved in Medium 199 for 2 h prior to addition of SGLT2 inhibitors, AMPK activators or IL-1β. Cell lysates were prepared, proteins resolved by SDS-PAGE and subjected to quantitative immunoblotting with the antibodies indicated as described previously^27,50^. Immunolabelled proteins were visualized using infrared dye-labelled secondary antibodies and an Odyssey Sa infrared imaging system (LiCor Biosciences UK Ltd, Cambridge, UK). Band density was quantitated with Image J software.

### Preparation of cell membrane fractions

Mouse kidneys were homogenized in 5 vol HES buffer (20 mmol/l Na HEPES-NaOH (pH 7.4), 1 mmol/l EDTA, 250 mmol/l sucrose; Roche complete protease inhibitor cocktail) with a Dounce homogenizer and centrifuged (7,050 × *g*, 20 min, 4 °C). The pellet was resuspended in HES buffer and layered on top of buffer containing 1.12 mol/l sucrose, prior to centrifugation in a swing-out rotor (25,000 × *g*, 60 min, 4 °C). Membranes were collected from the interface of the sucrose layer, diluted in HES buffer and centrifuged (150,000 × *g*, 60 min, 4 °C). The resultant plasma membrane-rich pellet was re-suspended in 0.2 ml HES buffer.

### Immunoprecipitation and assay of AMPK activity

AMPK was immunoprecipitated from cell lysates using a mixture of sheep anti-AMPKα1 and anti-AMPKα2 antibodies and assayed using the SAMS substrate peptide as previously described^27,50^.

### HUVEC proliferation assay

HUVECs (2 × 10^4^ cells/well) were allowed to attach to 96-well plates for 4 h in MV2 medium containing 2.5% or 5% (v/v) MV2 Supplement Mix (Promocell C-39226) containing serum and growth factors and subsequently incubated in the same medium with DMSO (vehicle) or SGLT2 inhibitors for 12 h. BrdU reagent was then added and cells further incubated for 24 h. BrdU incorporation was assessed by measuring A_485/520_ on a BMG Labtech FLUOstar Optima spectrophotometer microplate reader.

### HUVEC viability assay

HUVECs (2 × 10^4^ cells/well) were allowed to attach to 96-well plates for 4 h in MV2 medium containing 5% (v/v) MV2 Supplement Mix and subsequently incubated in the same medium with DMSO (vehicle) or SGLT2 inhibitors for 24 h. CellTiter 96® AQ_ueous_ One Solution Aqueous Cell solution was added 1.5 h prior to the end of incubation, at which point A_485_ was measured on a BMG Labtech FLUOstar Optima spectrophotometer microplate reader.

### Analysis of MCP-1, IL-6 and endothelin-1 secretion

Confluent monolayers of HUVECs or HAECs in 6-well plates were washed into medium 199 and preincubated in the presence or absence of A769662 or SGLT2 inhibitors for 30 min or 15 min respectively prior to stimulation with 10 ng/ml IL-1β for 6 h (MCP-1 and IL-6 secretion) or 24 h (endothelin-1 secretion) and the culture medium transferred to microcentrifuge tubes. Conditioned media were centrifuged (3,130 × *g*, 20 min, 4 °C and MCP-1, IL-6 or endothelin-1 levels assayed in supernatants using Quantikine ELISA kits as per the manufacturer’s instructions.

### 2-deoxyglucose uptake assay

HUVECs were incubated in serum-free Medium 199 for 2 hours, the media removed and replaced with Krebs-Ringer phosphate buffer (KRP) (130 mmol/l NaCl, 4.8 mmol/l KCl, 5 mmol/l NaH_2_PO_4_, 1.25 mmol/l MgSO_4_, 1.25 mmol/l CaCl_2_) containing 5 mmol/l glucose for 45 min. HUVECs were then incubated in glucose-free KRP in the presence or absence of SGLT2 inhibitors prior to the addition of 2-[^3^H]-deoxyglucose (50 µmol/l, 1 µCi/ml). Cytochalasin B-sensitive 2-deoxyglucose uptake was measured over 10 min as described previously^19^.

### U937 cell adhesion assay

HUVECs were grown to confluence on 24-well tissue culture plates and pre-incubated in the presence or absence of A769662, canagliflozin and/or SP600125 as indicated. Cells were subsequently stimulated in the presence or absence of 10 ng/ml IL-1β for 6 h. The medium was subsequently aspirated and HUVEC monolayers washed thoroughly with serum-free RPMI 1640 to remove activators/inhibitors prior to overlay with 1 × 10^5^ U937 cells/well in serum-free RPMI 1640. The cells were allowed to adhere for 1 h at 37 °C, the medium removed, and monolayers washed three times with serum-free RPMI 1640 to remove non-adherent U937 cells. Cells were fixed in PBS supplemented with 4% (w/v) paraformaldehyde and 5% (w/v) sucrose, and the number of adhered U937 cells per field of confluent HUVECs counted, with the investigator blinded as to the experimental conditions, on a Zeiss Axiovert 135 microscope with a X5 objective.

### RNA extraction and gene expression analysis

RNA was extracted from HAECs, HUVECs or HEK-293 cells using an RNeasy kit (Qiagen). Between 400–1000 ng of RNA was reverse-transcribed using the High Capacity cDNA Reverse Transcription kit (Applied Biosystems). qPCR was performed with an Applied Biosystems ABI-PRISM 7900HT Sequence Detection System. Gene expression was normalized to TATA binding protein (TBP) using QPCR master mix (Applied Biosystems) and the following TaqMan® Gene Expression Assays (Applied Biosystems): *TBP* (Hs00427620_m1), *SLC5A1* (SGLT1, Hs01573793_m1), *SLC5A2* (SGLT2, Hs00894642_m1), *IL6* (Hs00174131_m1) and *CCL2* (MCP-1, Hs00234140_m1).

### Assessment of cell surface E-Selectin, VCAM-1 and ICAM-1 levels

Confluent HAECs were incubated in the presence or absence of canagliflozin (10 μmol/l, 15 min) or A769662 (100 μmol/l, 30 min) prior to stimulation with 5 ng/ml IL-1β for 4 h. Cells were gently dislodged by trypsinisation, neutralised with complete MV2 culture medium and re-suspended in PBS supplemented with 1% (w/v) BSA (PBS-BSA). Cells were then incubated with saturating concentrations of phycoerythrin-labelled anti-ICAM-1, anti-VCAM-1 or anti-E-Selectin antibodies for 1 h, washed three times and resuspended in PBS-BSA. Cell surface adhesion molecule expression was evaluated in 10^4^ cells by flow cytometry using a FACSCalibur flow cytometer (BD Bioscience, Oxford, UK).

### Statistics

Results are expressed as mean ± SEM. Statistically significant differences were determined using a two-tail t-test, or ANOVA where appropriate, with *p* < 0.05 as significant using GraphPad Prism software.

### Data availability

The datasets generated during and/or analysed during the current study are available from the corresponding author on reasonable request.

## Electronic supplementary material

Supplementary data
