# Supplementary material for: Canagliflozin inhibits interleukin-1β-stimulated cytokine and chemokine secretion in vascular endothelial cells by AMP-activated protein kinase-dependent and -independent mechanisms
Source: Sci Rep. 2018 Mar 27;8:5276. doi: 10.1038/s41598-018-23420-4 (PMC5869674; doi:10.1038/s41598-018-23420-4)
Supplement: Supplementary file 1 — Supplementary data [file 41598_2018_23420_MOESM1_ESM.pdf]

Supplementary Information

Canagliflozin inhibits interleukin-1β-stimulated cytokine and chemokine secretion in vascular endothelial cells by AMP-activated protein kinase-dependent and -independent mechanisms.

Sarah J. Mancini[1], Daria Boyd[1], Omar J. Katwan[1,2], Anastasiya Strembitska[1], Tarek A. Almabrouk[1,3], Simon Kennedy[1], Timothy M. Palmer[4] and Ian P. Salt[1]*

[1]Institute of Cardiovascular and Medical Sciences, College of Medical, Veterinary and Life Sciences, University of Glasgow, Glasgow G12 8QQ, United Kingdom

[2]Department of Biochemistry, College of Medicine, University of Diyala, Baqubah, Iraq.

[3]Medical School, University of Zawia, Zawia, Libya

[4]School of Pharmacy and Medical Sciences, University of Bradford, Bradford, West Yorkshire, BD7 1DP, United Kingdom

SUPPLEMENTARY MATERIALS AND METHODS

Materials

Human aortic vascular smooth muscle cells and angiotensin II were from Sigma-Aldrich (Gillingham, UK). Compound 991 was synthesised by MRC Technology. Rabbit anti-phospho-AMPKα Thr172 (#2535), anti-AMPKα (#2532) and anti-p65 NFκB (#8242) antibodies were from New England Biolabs (Hitchin, Hertfordshire, UK). Rabbit anti-SGLT1 (#07-1417) antibodies were from Millipore (Watford, UK). Goat anti-rabbit Alexa Fluor 488 secondary antibodies were from Invitrogen (Paisley, UK). RedDot far-red nuclear stain was from Cambridge Bioscience (Cambridge, UK). All other reagents were from sources described previously[26,27,50].

Cell culture of human aortic vascular smooth muscle cells

Human aortic vascular smooth muscle cells (HAoVSMCs) were cultured in smooth muscle cell growth medium 2 (Promocell) and used for experiments between passages 3 and 6.

Preparation of HAoVSMC lysates, SDS PAGE and immunoblotting

HAoVSMCs were serum-starved in Medium 199 for 2 h prior to stimulation with the indicated concentrations of A769662, canagliflozin, angiotensin II and IL-1β for the indicated durations. Cell lysates were prepared, proteins resolved by SDS-PAGE and subjected to quantitative immunoblotting with the antibodies indicated as described previously[27,50].

Immunofluorescence confocal microscopy

Confluent layers of HAECs on glass coverslips were incubated in serum-free Medium 199 for 2 h and then KRH buffer (119 mmol/l NaCl, 20 mmol/l HEPES-NaOH (pH 7.4), 5 mmol/l $NaHCO_3$, 5 mmol/l KCl, 1.2 mmol/l $MgSO_4$, 1.2 mmol/l NaH_2PO_4, 2.5 mmol/l $CaCl_2$, 5 mmol/l glucose for 30 min prior to stimulation with canagliflozin, A769662 and/or IL-1β. Cells were fixed in 3% (w/v) paraformaldehyde at room temperature for 25 min and remaining paraformaldehyde quenched by two washes with PBS supplemented with 20 mmol/l glycine. Coverslips were then washed twice in PBS and incubated in PBS containing 2% (w/v) BSA, 0.1% (w/v) Triton X-100, 20 mmol/l glycine for 10 min. Coverslips were washed a further two times with PBS prior to blocking in IF buffer (PBS supplemented with 2% (w/v) BSA, 20 mmol/l glycine, 0.1% (v/v) goat serum) for 20 min.

Coverslips were then incubated in rabbit anti-p65 NFκB antibodies in IF buffer for 60 min, washed in IF buffer four times and further incubated in Alexa Fluor® 488-conjugated goat anti-rabbit IgG secondary antibodies and RedDot diluted in IF buffer for 30 min. Cells were washed four times in IF buffer, once in PBS and mounted on microscope slides prior to visualisation on a Zeiss LSM 5 Pascal Exciter laser scanning microscope.

Statistics

Results are expressed as mean ± SEM. Statistically significant differences were determined using a two-tail t-test, or ANOVA where appropriate, with $p < 0.05$ as significant using GraphPad Prism software.

Data availability

The datasets generated during and/or analysed during the current study are available from the corresponding author on reasonable request.

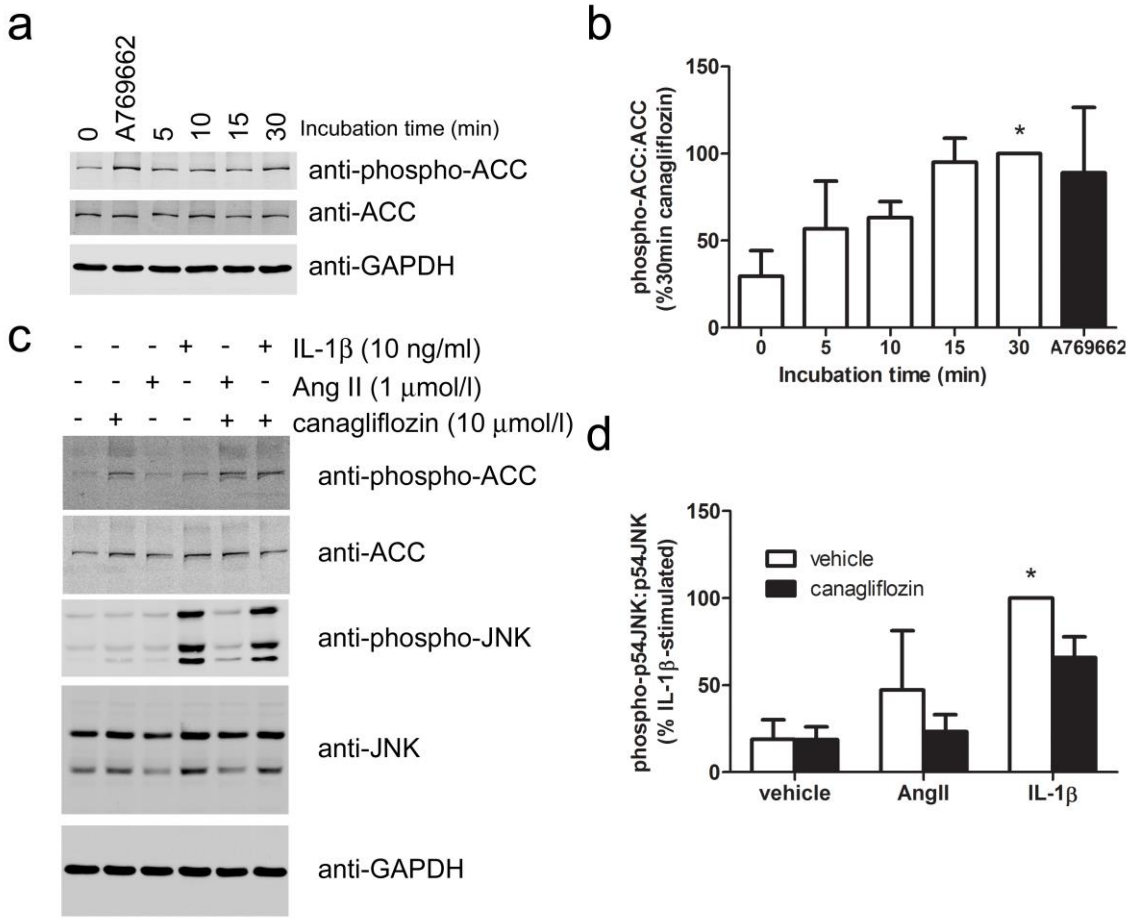

Supplementary Figure S1: Canagliflozin stimulates AMPK without altering IL-1β-stimulated JNK phosphorylation in HAoVSMCs

HAoVSMCs were (a) incubated in the presence or absence of canagliflozin (10 μmol/l) for the indicated times or 100 μmol/l A769662 for 30 min or (b) canagliflozin (10 μmol/l, 30 min) prior to stimulation with IL-1β (10 ng/ml, 10 min) or angiotensin II (1 μmol/l, 10 min, Ang II) and lysates prepared. Lysate proteins were resolved by SDS-PAGE and subjected to immunoblotting with the antibodies indicated. (a, c) Representative immunoblots, repeated on two further occasions with similar results are shown and have been cropped, with the full-length immunoblots shown in Supplementary Figure S12. (b, d) Densitometric analysis of (b) ACC and (d) JNK (p54) phosphorylation normalised to respective total levels from three independent experiments. *p<0.05 vs vehicle (ANOVA).

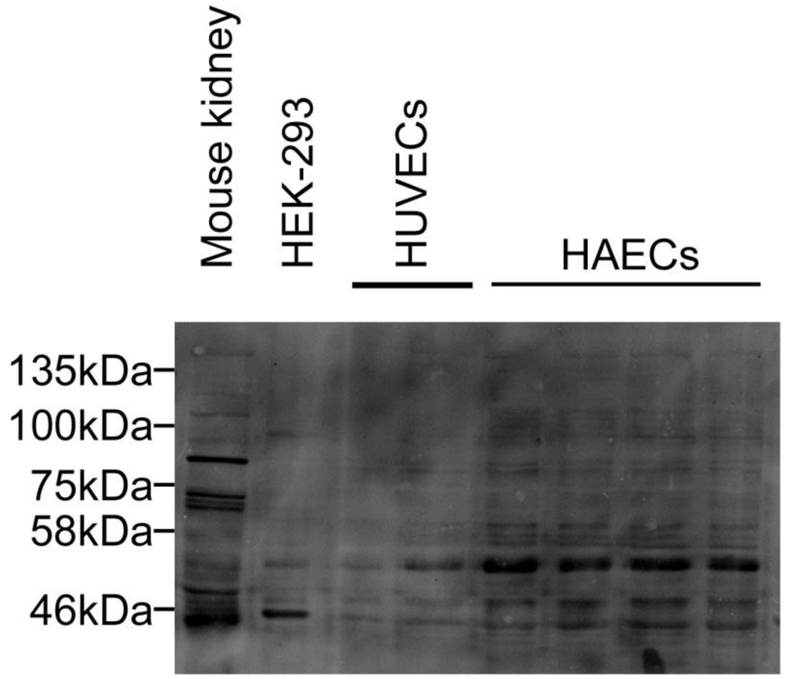

Supplementary Figure S2: Anti-SGLT1 immunoreactivity in HUVECs and HAECs
Cell lysates from HUVECs, HAECs and HEK-293 cells and mouse kidney membranes were resolved by SDS-PAGE and immunoblotted with anti-SGLT1 antibodies. A representative image is shown and has been cropped, with the full-length immunoblot shown in Supplementary Figure S7.

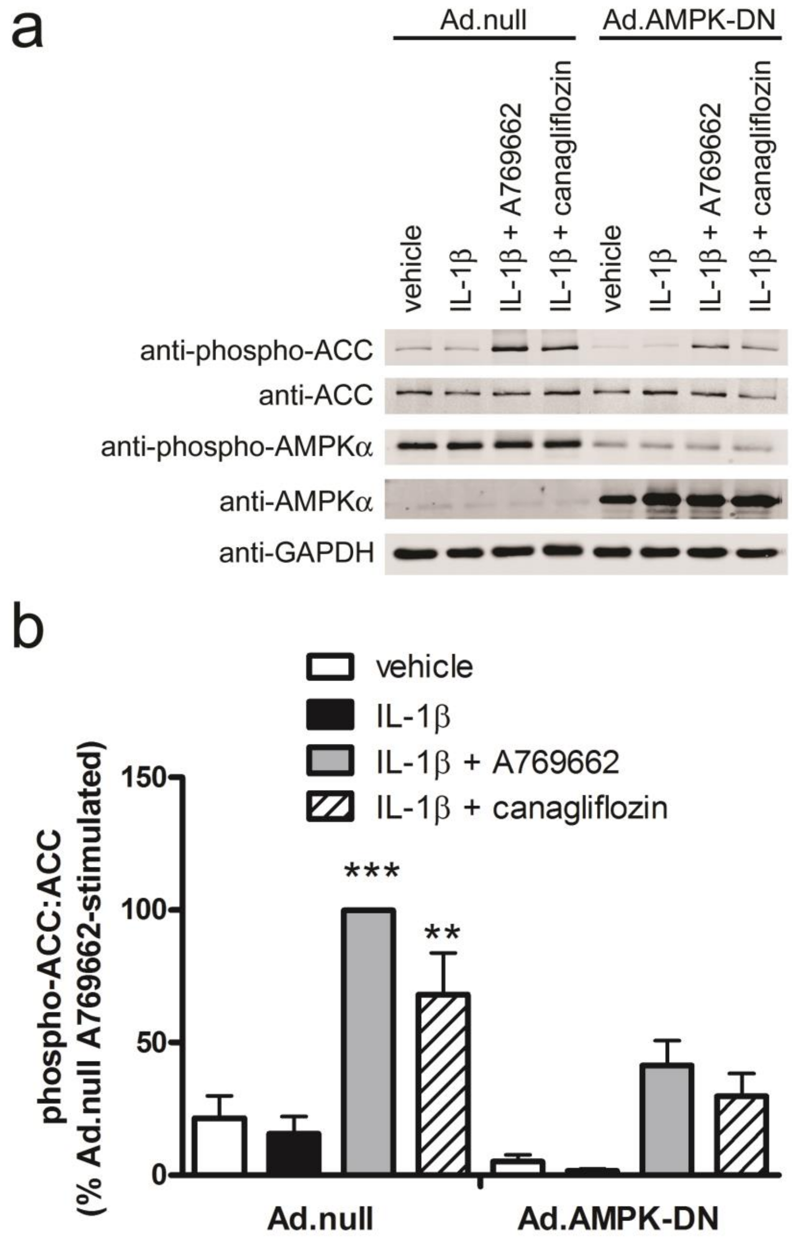

Supplementary Figure S3: Suppression of HAEC AMPK activity using adenoviruses expressing a dominant negative mutant AMPK

HAECs were infected with 100 pfu/cell Ad.null or Ad.AMPK-DN for 24 h and preincubated in the presence or absence of canagliflozin (10 µmol/l, 15 min) or A769662 (100 µmol/l, 30 min) prior to stimulation with IL-1β (10 ng/ml) for 6 h. Lysates were prepared, resolved by SDS-PAGE and immunoblotted with the indicated antibodies. (a) Representative images are shown from three independent experiments and have been cropped, with the full-length immunoblots shown in Supplementary Figure S13, with phosphorylation of ACC quantified in (b). $^{**}p<0.01$, $^{***}p<0.001$ relative to IL-1β alone (ANOVA).

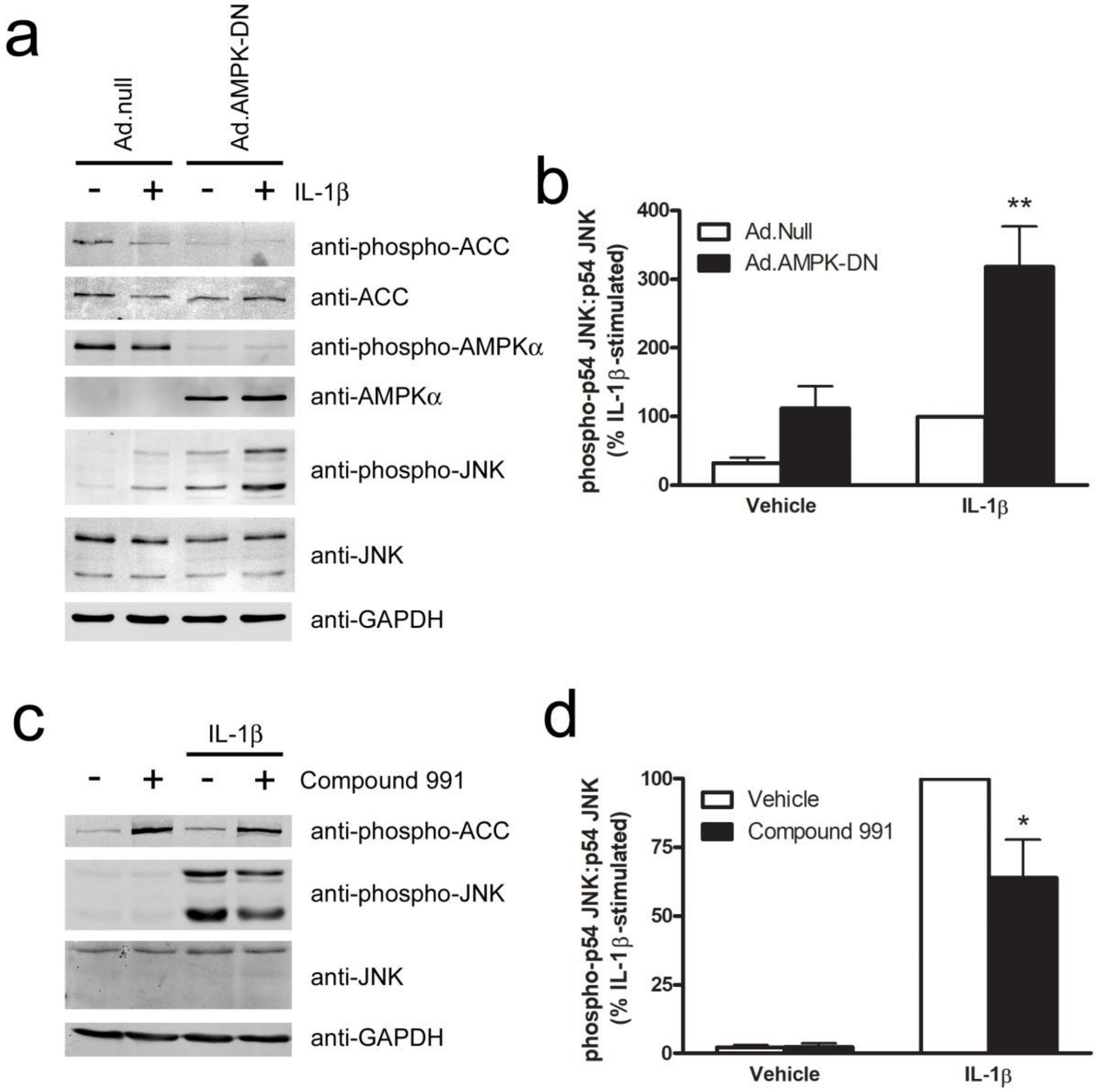

Supplementary Figure S4: IL-1β-stimulated JNK phosphorylation is inhibited by compound 991 and increased by AMPK inhibition in endothelial cells

(a, b) HAECs were infected with 100 pfu/cell Ad.null or Ad.AMPK-DN for 24 h and preincubated in the presence or absence of IL-1β (10 ng/ml) for 6 h. (c, d) HUVECs were incubated in the presence or absence of compound 991 (1 μmol/l, 20 min) prior to stimulation with IL-1β (10 ng/ml, 15 min). Cell lysates were prepared, resolved by SDS-PAGE and immunoblotted with the indicated antibodies. (a, c) Representative immunoblots are shown from four independent experiments and have been cropped, with the full-length immunoblots shown in Supplementary Figure S14. (b, d) Densitometric analysis of JNK (p54) phosphorylation normalised to respective total levels from four independent experiments. *p<0.05 vs absence of Compound 991, **p<0.01 relative to Ad.null-infected cells (ANOVA).

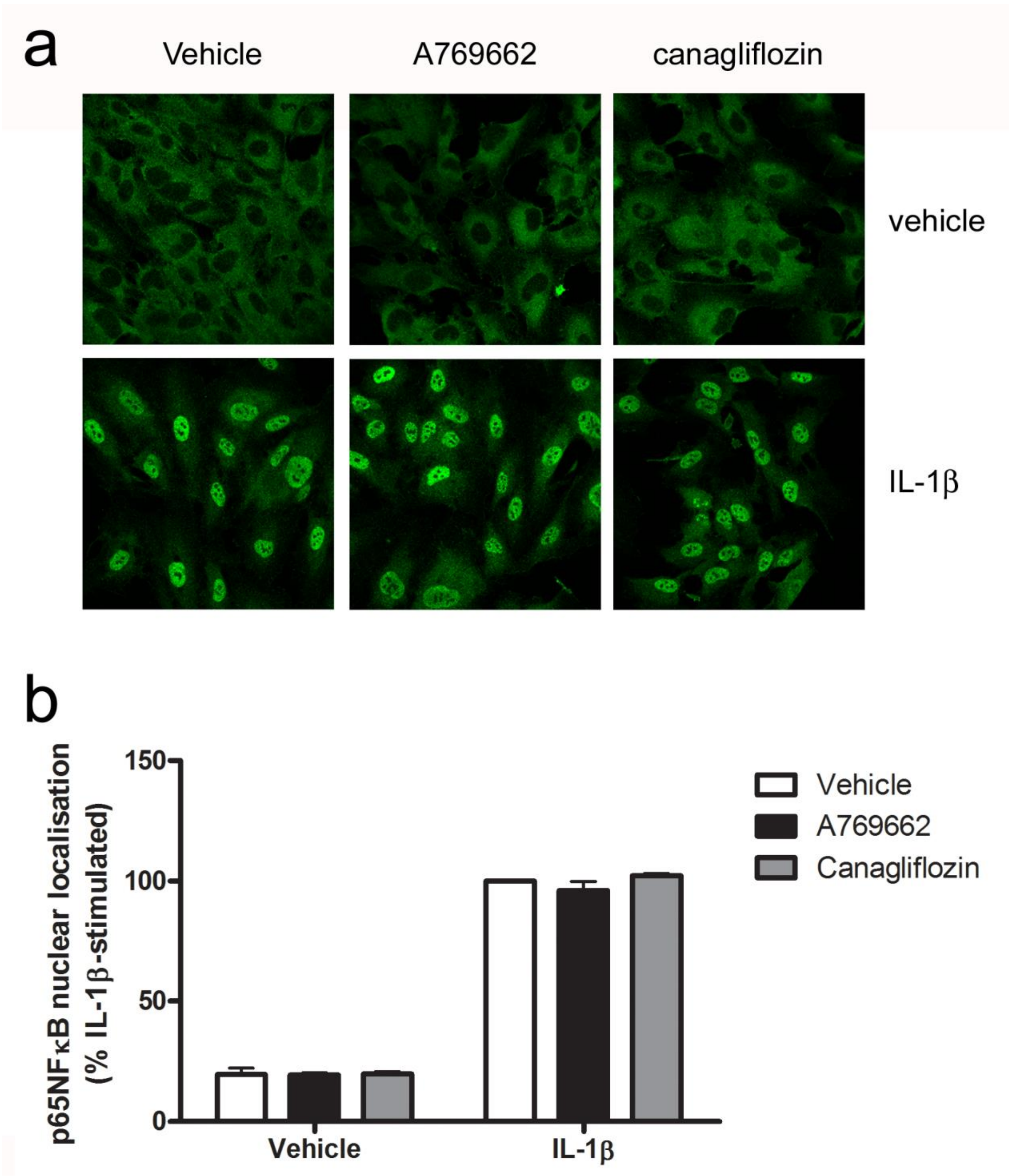

Supplementary Figure S5: Canagliflozin and A769662 have no effect on IL-1β-stimulated p65 NFκB nuclear translocation in HAECs.

HAECs were incubated with IL-1β (5 ng/ml, 15 min) following preincubation in the presence or absence of canagliflozin (10 µmol/l, 15 min) or A769662 (100 µmol/l, 30 min) and NFκB (p65) localisation assessed by confocal fluorescence microscopy. (a) Representative images are shown. (b) Densitometric quantification of nuclear p65 NFκB fluorescence. All data are presented as % IL-1β-stimulated nuclear fluorescence from three independent experiments with > 50 cells analysed for each treatment in each experiment.

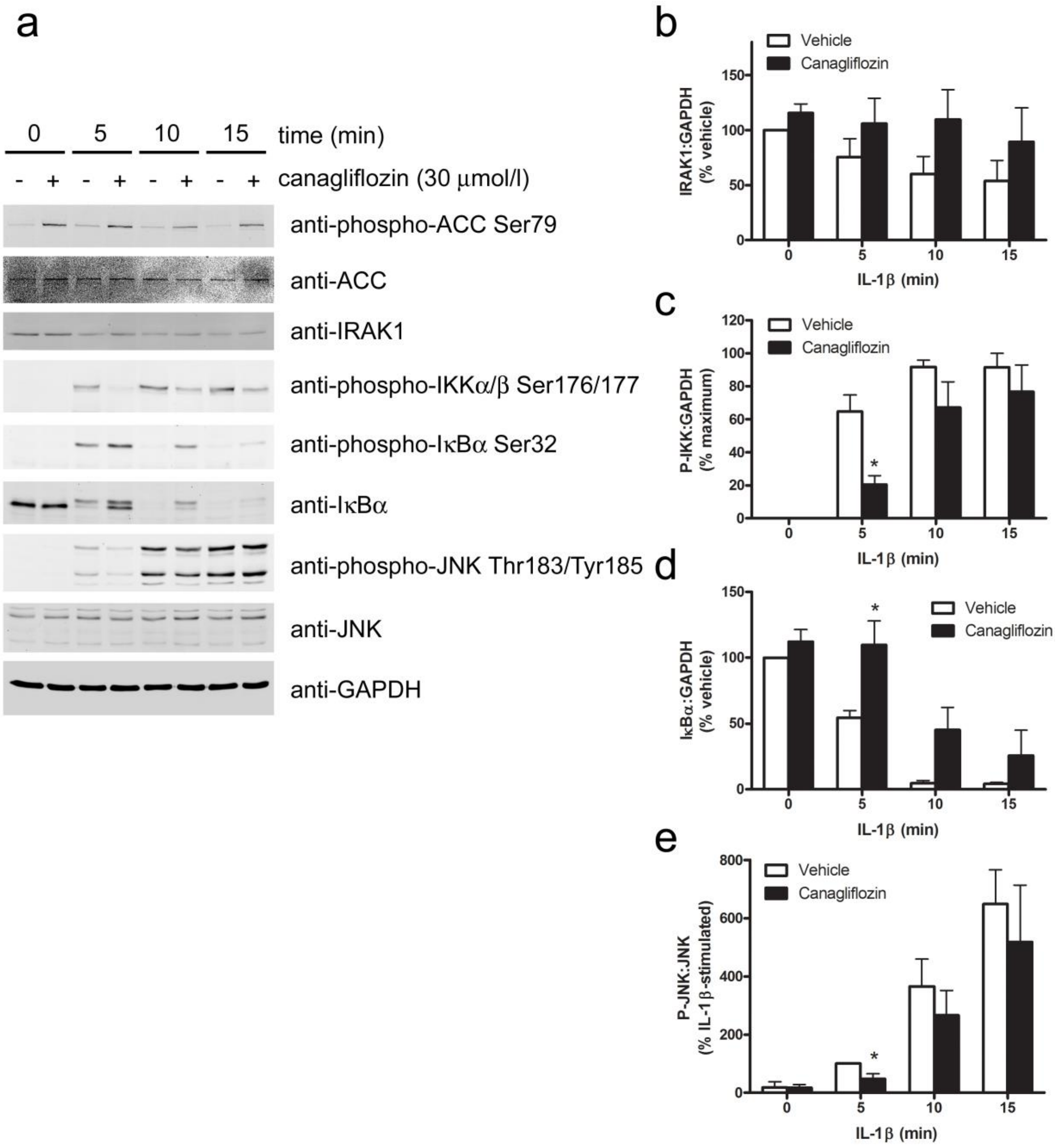

Supplementary Figure S6: High concentrations of canagliflozin inhibit initial IL-1β proinflammatory signalling pathways

HUVECs were incubated for 15 min in the presence or absence of canagliflozin (30 μmol/l) prior to stimulation with IL-1β (10 ng/ml) for the times indicated and lysates prepared. Lysate proteins were resolved by SDS-PAGE and subjected to immunoblotting with the antibodies indicated. (a) Representative immunoblots are shown and have been cropped, with the full-length immunoblots shown in Supplementary Figure S15 (b-e) Densitometric analysis of (b) IRAK1, (c) phospho-IKK, (d) IκB relative to GAPDH or (e) JNK (p54) phosphorylation normalised to respective total p54 JNK levels from three (b-d) or four (e) independent experiments. $^{*}p<0.05$ vs absence of canagliflozin (ANOVA).

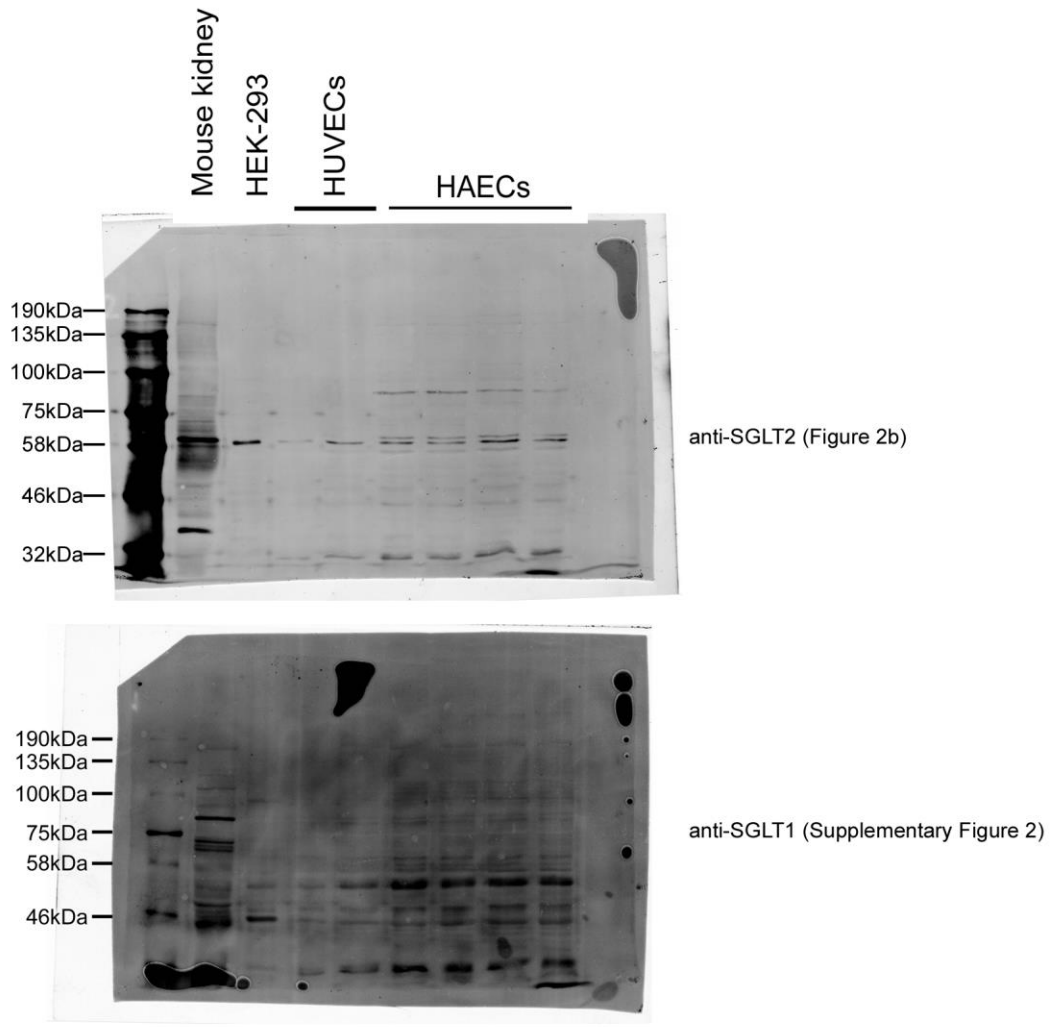

Supplementary Figure S7: Full size immunoblots of Figure 2b and Supplementary Figure S2.

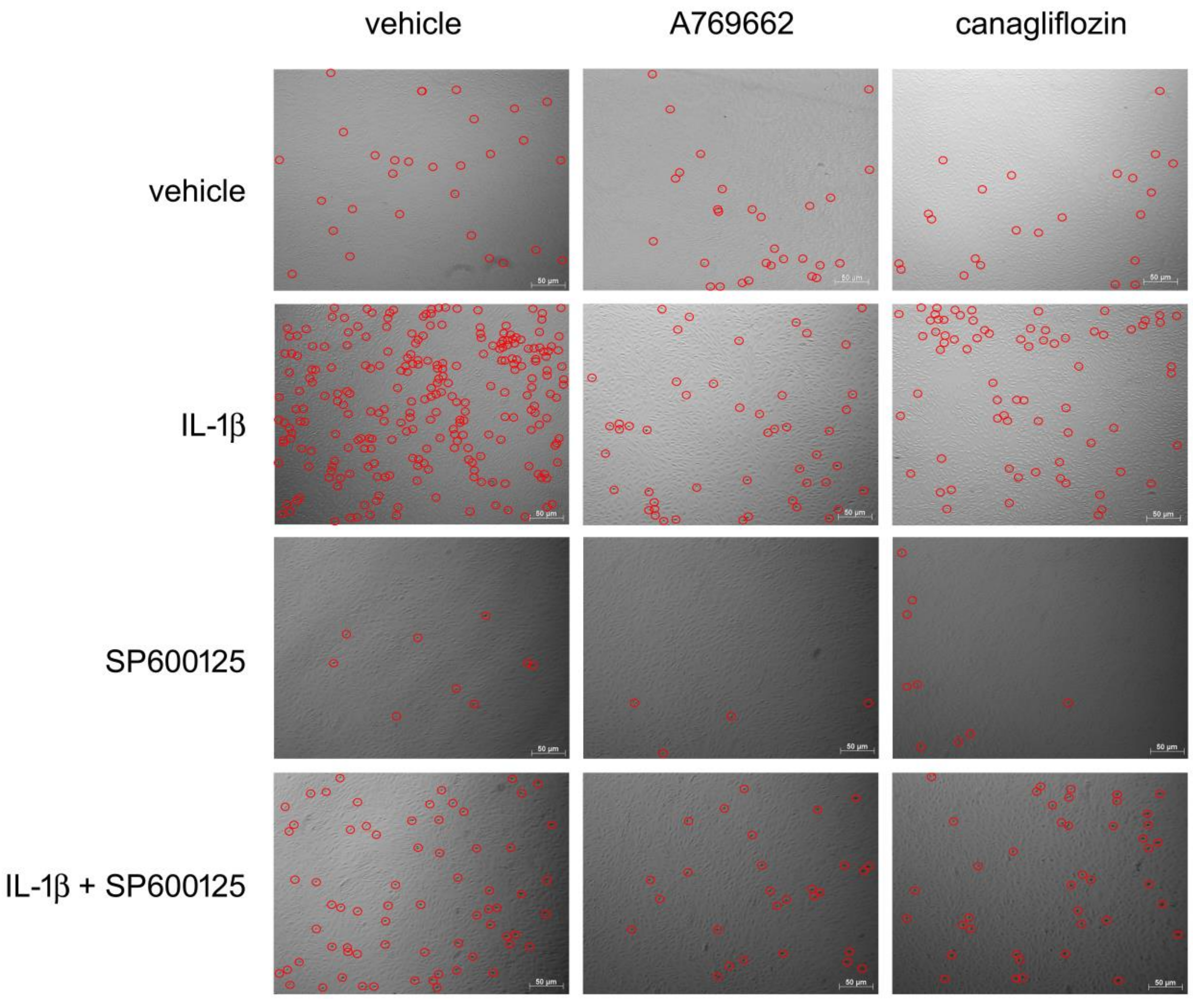

Supplementary Figure S8: Representative micrographs of U937 cell adhesion to HUVECs quantified in Figure 5a.

Representative micrographs of each experimental condition are shown (scale bar = 50 µm), obtained using a Zeiss Axiovert 135 microscope with an X5 objective, with adhered U937 cells indicated by red circles.

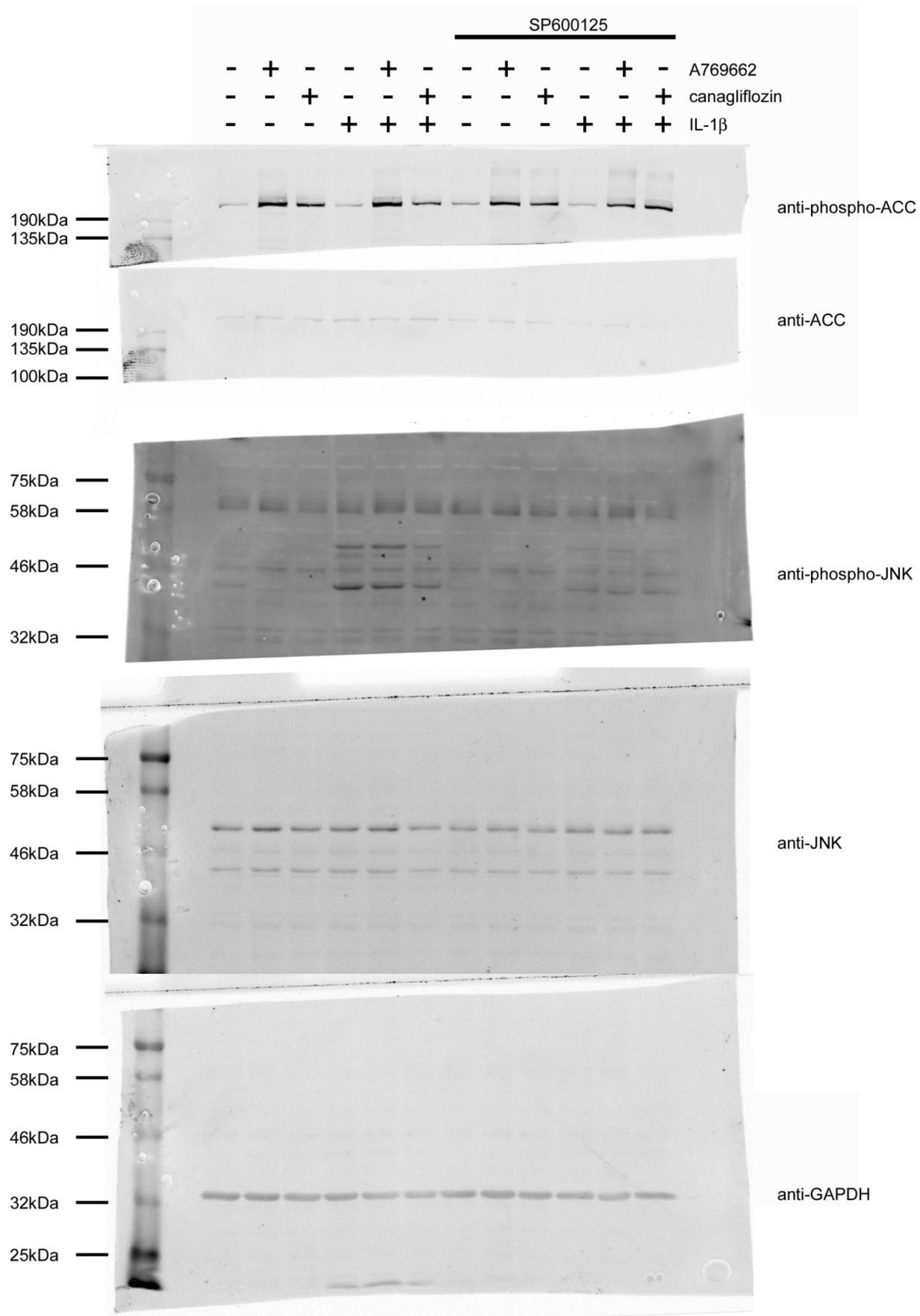

Supplementary Figure S9: Full size immunoblots of Figure 5b.

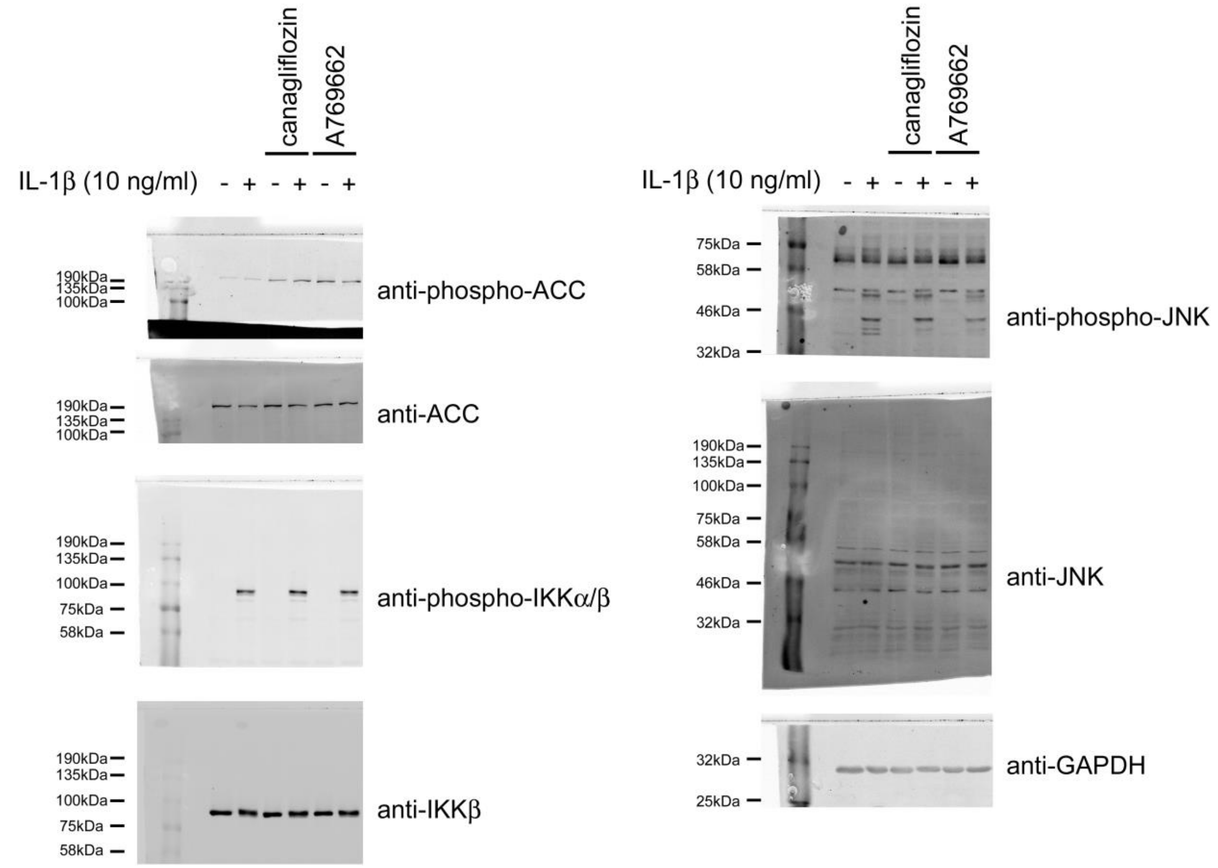

Supplementary Figure S10: Full size immunoblots of Figure 6a:

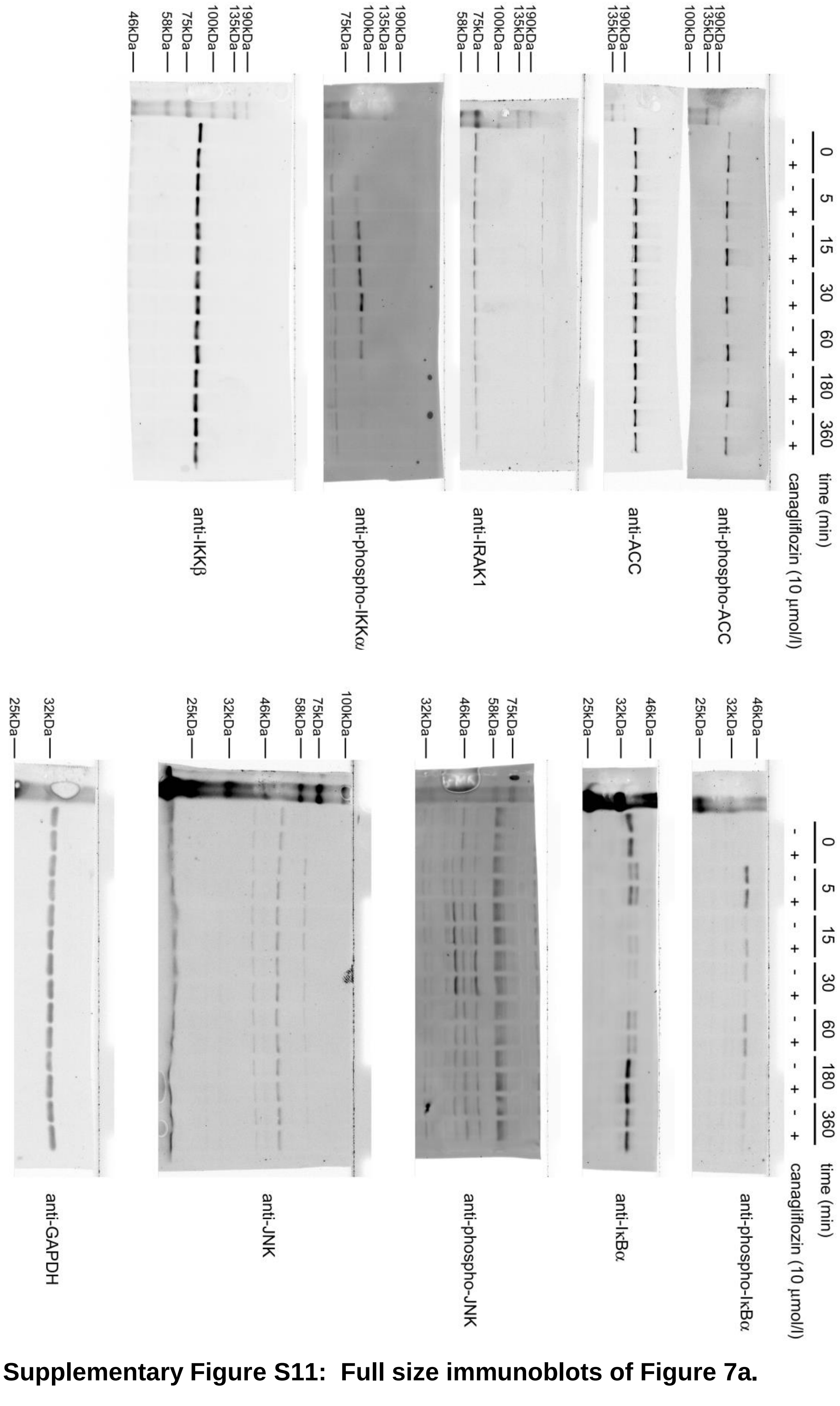

Supplementary Figure S11: Full size immunoblots of Figure 7a.

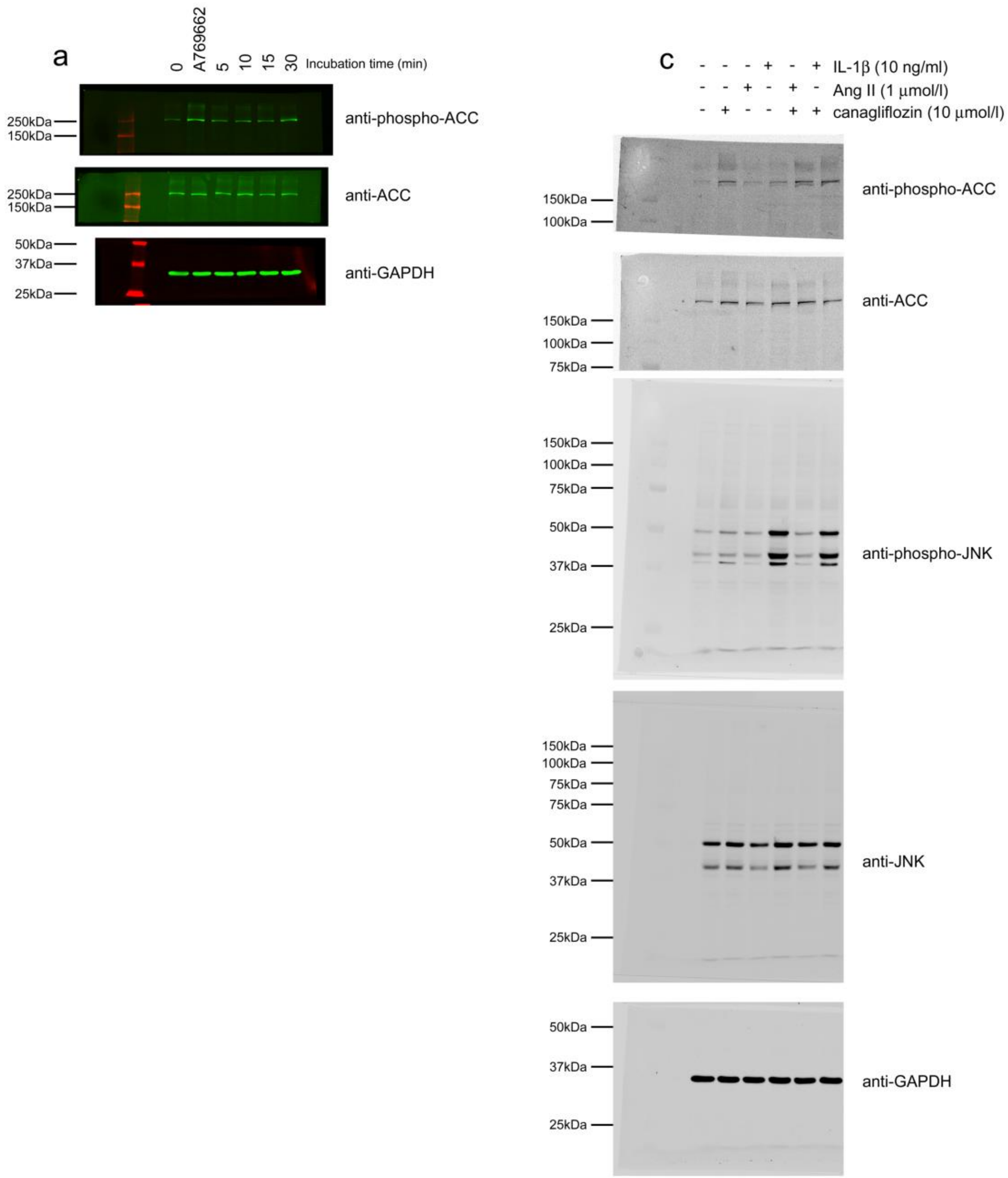

Supplementary Figure S12: Full size immunoblots of Supplementary Figure S1a and S1c.

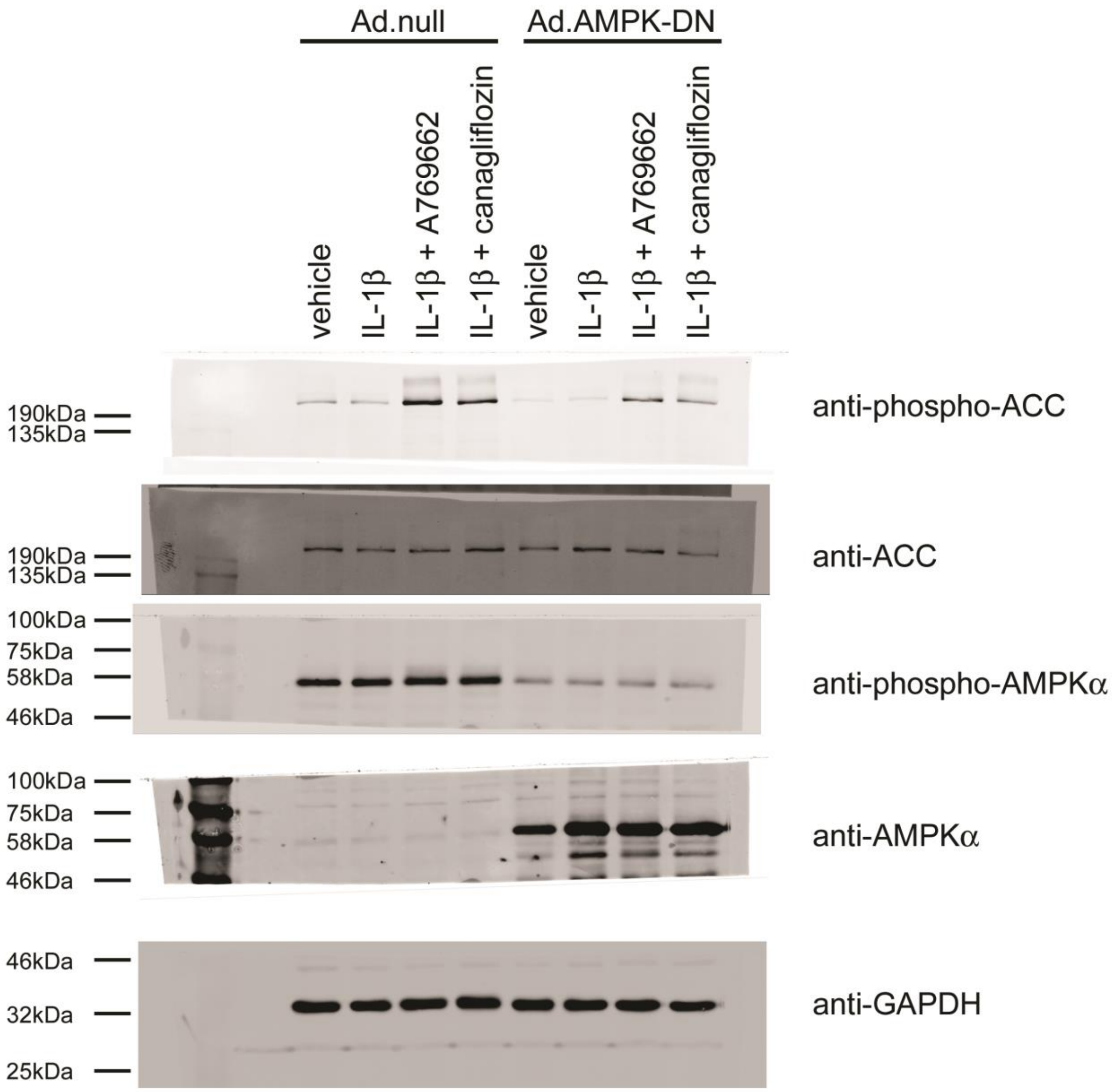

Supplementary Figure S13: Full size immunoblots of Supplementary Figure S2a.

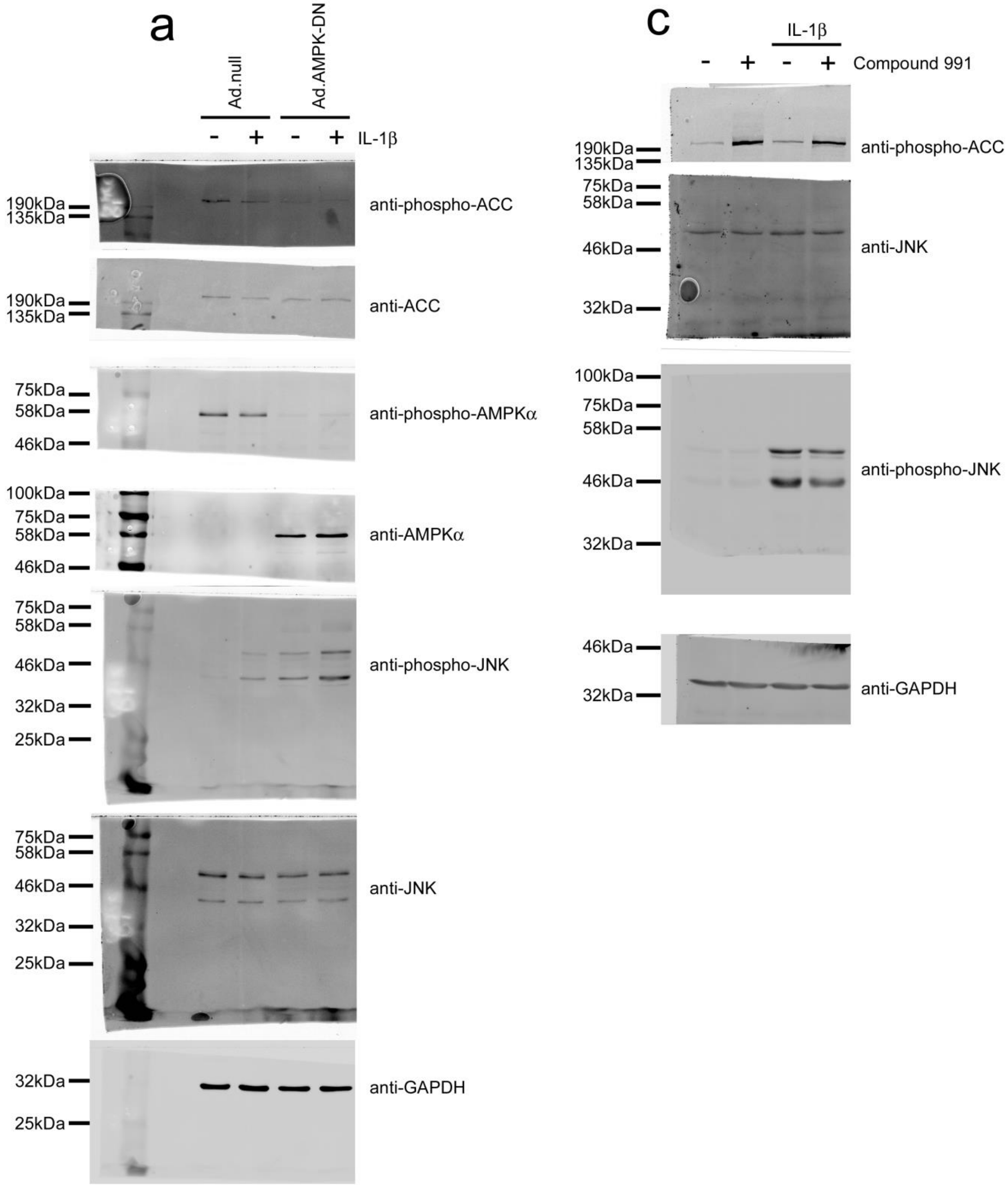

Supplementary Figure S14: Full size immunoblots of Supplementary Figure S3a and S3c.

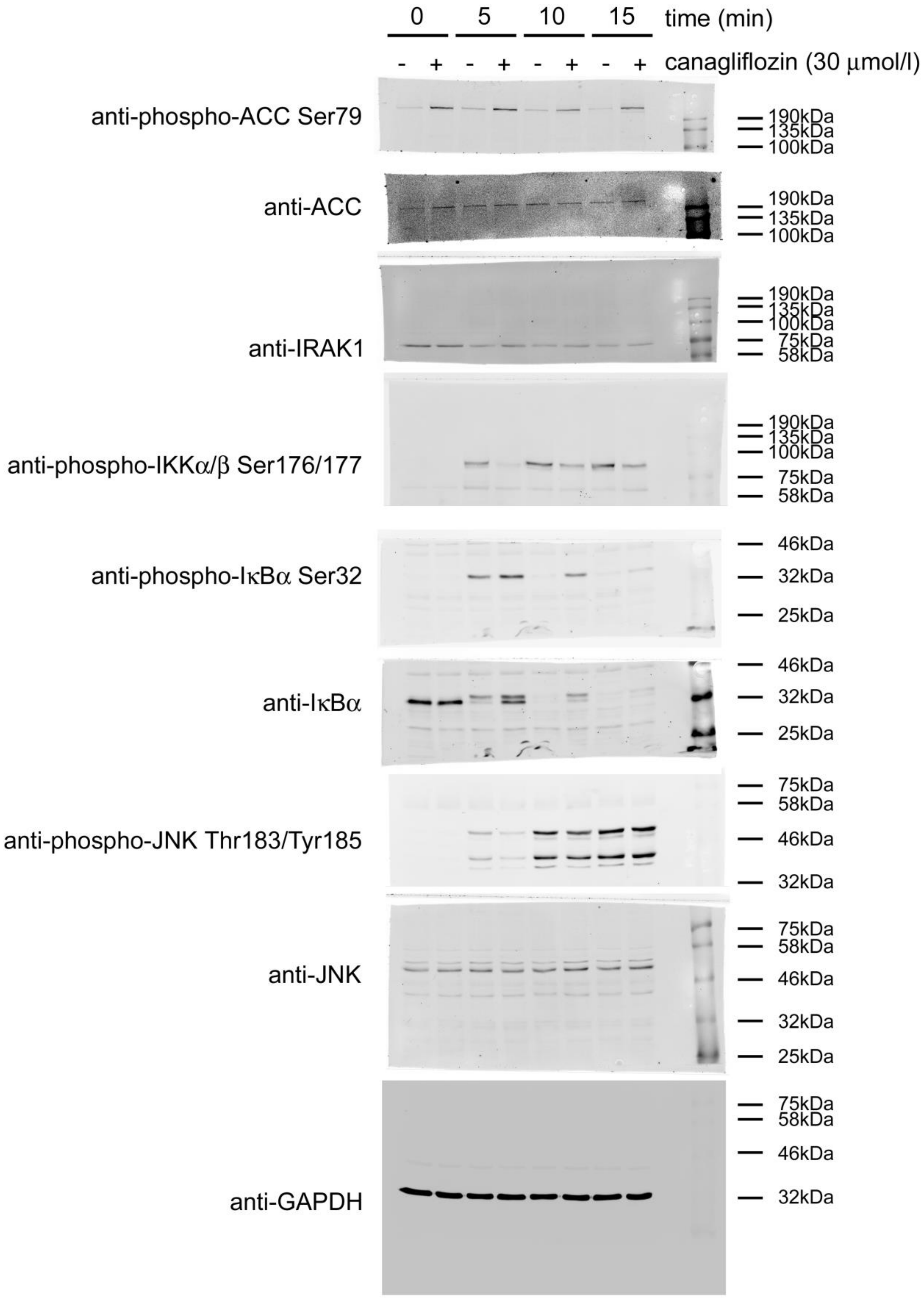

Supplementary Figure S15: Full size immunoblots of Supplementary Figure S6a.
